# An Automated Patient Self-Monitoring System to Reduce Health Care System Burden During the COVID-19 Pandemic in Malaysia: Development and Implementation Study

**DOI:** 10.2196/23427

**Published:** 2021-02-26

**Authors:** Hooi Min Lim, Chin Hai Teo, Chirk Jenn Ng, Thiam Kian Chiew, Wei Leik Ng, Adina Abdullah, Haireen Abdul Hadi, Chee Sun Liew, Chee Seng Chan

**Affiliations:** 1 Department of Primary Care Medicine University of Malaya Medical Centre Kuala Lumpur Malaysia; 2 eHealth Unit Faculty of Medicine University of Malaya Kuala Lumpur Malaysia; 3 Dean's Office Faculty of Medicine University of Malaya Kuala Lumpur Malaysia; 4 Department of Primary Care Medicine Faculty of Medicine University of Malaya Kuala Lumpur Malaysia; 5 Department of Software Engineering Faculty of Computer Science and Information Technology University of Malaya Kuala Lumpur Malaysia; 6 Department of Computer System and Technology Faculty of Computer Science and Information Technology University of Malaya Kuala Lumpur Malaysia; 7 Department of Artificial Intelligence Faculty of Computer Science and Information Technology University of Malaya Kuala Lumpur Malaysia

**Keywords:** COVID-19, coronavirus disease, home monitoring, symptom monitoring, system, teleconsultation, development, eHealth, digital health, mHealth, health services research, telesurveillance, infectious disease, app

## Abstract

**Background:**

During the COVID-19 pandemic, there was an urgent need to develop an automated COVID-19 symptom monitoring system to reduce the burden on the health care system and to provide better self-monitoring at home.

**Objective:**

This paper aimed to describe the development process of the COVID-19 Symptom Monitoring System (CoSMoS), which consists of a self-monitoring, algorithm-based Telegram bot and a teleconsultation system. We describe all the essential steps from the clinical perspective and our technical approach in designing, developing, and integrating the system into clinical practice during the COVID-19 pandemic as well as lessons learned from this development process.

**Methods:**

CoSMoS was developed in three phases: (1) requirement formation to identify clinical problems and to draft the clinical algorithm, (2) development testing iteration using the agile software development method, and (3) integration into clinical practice to design an effective clinical workflow using repeated simulations and role-playing.

**Results:**

We completed the development of CoSMoS in 19 days. In Phase 1 (ie, requirement formation), we identified three main functions: a daily automated reminder system for patients to self-check their symptoms, a safe patient risk assessment to guide patients in clinical decision making, and an active telemonitoring system with real-time phone consultations. The system architecture of CoSMoS involved five components: Telegram instant messaging, a clinician dashboard, system administration (ie, back end), a database, and development and operations infrastructure. The integration of CoSMoS into clinical practice involved the consideration of COVID-19 infectivity and patient safety.

**Conclusions:**

This study demonstrated that developing a COVID-19 symptom monitoring system within a short time during a pandemic is feasible using the agile development method. Time factors and communication between the technical and clinical teams were the main challenges in the development process. The development process and lessons learned from this study can guide the future development of digital monitoring systems during the next pandemic, especially in developing countries.

## Introduction

To combat the COVID-19 pandemic, digital technology has been used extensively for health information dissemination, contact tracing [1], population surveillance, and forecasting modeling [2]. It provides a platform for real-time updates of COVID-19 cases, performs modeling studies to forecast COVID-19 disease activity, and disseminates public health education [3]. However, there are issues related to technology use during a pandemic; there are occasions when urgency supersedes accuracy, which may compromise patient and public safety. The accuracy and security of a technology must be considered carefully and scrutinized systematically to ensure patients’ safety. Also, false information may cause unnecessary alarm and chaos in the population or may fail in the detection or monitoring of those at risk of infection [4]. Privacy and data protection concerns have been raised with large-scale digital data collection during the pandemic [5]. Therefore, there is a need for research, risk assessments, and pilot studies before technologies roll out to the public in order to avoid harms.

During the COVID-19 pandemic, several COVID-19 remote monitoring tools were developed and rolled out to reduce the burden on health care systems and to free up hospital spaces. In France, Covidom, a web-based application, was used as a remote surveillance tool for COVID-19 patients with mild to moderate symptoms in order to preserve medical resources for more severe patients and limit in-person interactions during the pandemic [6]. COVID-19 symptom monitoring tools could potentially reduce the burden on the health care system. A practical and systematic structure is required to link digital monitoring tools with teleconsultation and local health care service systems. However, a detailed description of the developmental process and practical operational steps in developing a digital monitoring system for urgent use in a pandemic was lacking in the literature.

COVID-19 cases in Malaysia have increased since March 2020, with a total number of 70,236 confirmed COVID-19 cases as of December 5, 2020. It is estimated that for every positive COVID-19 patient diagnosed, there are 16 other suspected COVID-19 patients (ie, patients under investigation [PUIs]) and asymptomatic close contacts to be taken care of in Malaysia. These patients were quarantined and required daily follow-up [7]. In Malaysia, PUIs and close contacts of a confirmed case, who was fit to be discharged home, were given a home surveillance tool that included a checkbox list of COVID-19 symptoms [8]. Public health officers from the district health offices would call each patient via phone to monitor their symptoms daily for up to 14 days. This manual surveillance work is time-consuming and labor-intensive. Hence, there is an urgent need to use digital technology to monitor PUIs and close contacts during COVID-19 to reduce the burden on the health care system in Malaysia.

Conventionally, digital health systems are developed based on software development life cycle models, such as the waterfall, spiral, V-shaped, and rapid application development models [9]. In developing our COVID-19 Symptom Monitoring System (CoSMoS), an automated self-monitoring system that is the focus of this paper, the agile model was preferred because it uses an adaptive approach that easily adapts to changing requirements. This characteristic is important because the guidelines and clinical evidence of COVID-19 were changing rapidly, especially in the early phase of the pandemic, which would change the requirements, expressed as user stories, during the development. The agile method is more appropriate and feasible for developing a digital health app within a short period during a pandemic.

The development of CoSMoS was a time-intensive process requiring commitment on the part of the experts; in this context, that includes the expert in digital software development and the health care professionals who were actively involved in providing care during the pandemic. This paper aims to describe the development process of a COVID-19 monitoring system, CoSMoS, using the agile model within a short period during the COVID-19 pandemic as well as the lessons learned.

## Methods

### Overarching Development Framework and Phases

The agile software development life cycle was used to develop CoSMoS because it was urgently needed for patients suspected of having COVID-19. The development of CoSMoS was divided into three phases: requirement formation, development testing iteration, and integration into clinical practice. All requirements, development, and testing work were conducted online via Zoom, WhatsApp, Slack, Trello, and email. No physical meetings were held due to the COVID-19 movement control order (ie, lockdown) in Malaysia. The whole development process took 19 days, from study inception on March 21, 2020, to the launch of CoSMoS on April 9, 2020. The detailed timeline is shown in Multimedia Appendix 1. This study was approved by the Medical Research Ethics Committee of the University of Malaya Medical Centre (UMMC) (MECID No. 202043-8434).

### Development and Research Team

An interdisciplinary team-based approach, which has been commonly used for mobile health solutions in the literature, was adapted for the development of CoSMoS [10]. The core team, which was in charge of the overall study inception, development, and execution, comprised primary care physicians (n=6), computer science academicians (n=3), a patient advocate (n=1), and an eHealth research fellow (n=1). The clinical team (ie, six primary care physicians and an eHealth research fellow) was responsible for the content of CoSMoS and the integration of CoSMoS into clinical practice, including the workflow, monitoring, and teleconsultation. The technical team, which was in charge of the system development, consisted of the three computer science academicians, an eHealth research fellow, 26 students and graduates from the Faculty of Computer Science and Information Technology, and one user experience designer. The technical development process was led by an associate professor in computer science, who is an expert in software development and the agile model; this team member oversaw the development process; designed the system architecture, task, and workforce management; and supervised the developers. A high number of technical team members volunteered to develop CoSMoS so they could play a part contributing to combating the pandemic. The eHealth research fellow straddled across all teams and acted as the project coordinator between the clinical and technical teams. There were two postgraduate clinical master’s students in primary care medicine involved in integrating CoSMoS into clinical practice.

### Phase 1: Requirement Formation

#### Problem Identification and Requirements

The core team first met via Zoom to explore the problems in depth. This discussion was led by the clinical team, which consisted of a panel of seven experts in primary care health services, infectious disease, and health informatics. The panel obtained expert input from the district health officials and infectious control team from UMMC to have a clearer picture of the current COVID-19 situation.

The objectives of this discussion were to (1) identify the major problems in the process of monitoring PUIs and close contacts at home and the limitations of the existing system, (2) identify areas in which digital technology could help to reduce the burden on health care workers (HCWs) and provide a better home monitoring service, and (3) identify the target users of CoSMoS.

The problems identified were then converted to the requirements of the system, including the functions and features. The integration of CoSMoS into the existing clinical workflow was also discussed.

#### Algorithm and Content Drafting

With the list of requirements at hand, the clinical team started to draft the content and algorithm of CoSMoS. The clinical expert panel developed the clinical decision-making algorithm based on the World Health Organization and the Malaysian Ministry of Health guidelines of COVID-19 monitoring [7,8]. A decision tree method was used to develop the clinical algorithm, establish whether a patient was safe to continue monitoring at home, and establish whether a patient should call HCWs for teleconsultation [11]. The panel selected the variables to be included in the decision tree and assessed the importance of each variable in clinical decision making. We also considered the selection of clinical relevance variables when developing the algorithm. After the variables were selected, a decision node was determined, representing a choice that would divide the patients into asymptomatic or symptomatic. An *if-then-else* rule was used to create a decision tree pathway. The clinical team tested the algorithm using different clinical scenarios and revised the algorithm iteratively.

### Phase 2: Development Testing Iteration

#### Overview

The requirements and the contents drafted were presented to the technical team. The technical team explored potential solutions to achieve the requirements. The technical team reviewed several software development platforms and tools and made selections based on their suitability to deliver the solution; availability of development resources, including the team’s existing skill sets; security; affordability for the end users; and cost.

#### Continuous Integration and Continuous Deployment

The development of the CoSMoS system involved several short cycles comprising the following activities: elicit requirements, develop and test, review and feedback, and revise requirements. The continuous integration and continuous deployment (CI/CD) pipeline was used to enable swift flow from one activity to the next within a cycle, uninterrupted integration of deliverables (ie, software codes) of a cycle with deliverables of previous cycles, continuous testing by the core team, and deployment of the tested system into the production environment. CI/CD has two environments: staging (ie, nonproduction) and production. Any changes requested are first pushed to the staging environment for testing. Once the revision has been tested and finalized, it is swiftly pushed to the production environment.

#### Iterative Development Testing Cycles

Among the technical team members, there were nine developers focusing on testing before CoSMoS was tested by the core team members in a daily manner. The core team members reviewed CoSMoS (ie, the system’s Telegram bot and dashboard) in terms of its utility (eg, accuracy of the algorithm, the wording of the content, timing of daily ping messages, and correctness of patient categorization in the dashboard) and usability (eg, button size and position, layout, and the flow of content). The change requests with screenshots were compiled in a Word document and sent to the technical team, who would implement the changes in the evening. Managing change requests was crucial in ensuring that the requirements of CoSMoS were properly documented and updated.

### Phase 3: Integration Into Clinical Practice

CoSMoS was presented to the stakeholders, including the primary care clinic management team and clinicians, the hospital’s COVID-19 task force committee, and the emergency department. Live demonstrations of the system were used during these presentations, and the stakeholders could test out the CoSMoS Telegram bot. Feedback from the stakeholders on the utility, usability, and feasibility of the system was collected to further improve the system. Medicolegal aspects of the clinical implication and teleconsultation were discussed.

Repeated simulations with different clinical workflows were tested to identify the most effective and suitable clinical workflow for CoSMoS integration. Role-playing with clinic staff was done to test out the CoSMoS workflow in the actual clinic setting. Barriers and weaknesses in the workflow were identified. Careful consideration was done to reduce the physical contact between HCWs and patients with suspected COVID-19. We finalized the clinical workflow for the integration of CoSMoS after several rounds of testing. Training was conducted for all clinic staff, including the system’s objectives, functions, features, and operational guides; how to integrate CoSMoS into the existing workflow; the teleconsultation guide; and possible issues. Simulations were done during training to ensure familiarity with the new workflow. We installed the CoSMoS dashboard onto the desktop computers in the clinic. The doctors used designated smartphones with the CoSMoS Telegram bot installed for monitoring and teleconsultation. A technical help desk was set up to assist the doctors should any technical issues arise.

Records of patients recruited for CoSMoS will be kept in the hospital electronic medical record system and the CoSMoS system. These data will be kept confidential. Only the researchers, research assistants, and CoSMoS doctors have access to the medical record system, using designated log-in credentials. Data are transmitted over the https secure communication protocol and stored on cloud storage with adequate security protection.

## Results

### Phase 1: Requirement Formation

#### Problem Identification and Requirements

The expert panel identified the target users and sites for CoSMoS. The target users were the PUIs and close contacts who attended the primary care clinic and emergency department in the UMMC and the health care providers who used CoSMoS to monitor the patients. The expert panel identified four main problems in the existing home monitoring system for PUIs and close contacts that could be solved by digital innovation (see Table 1).

**Table 1:** Problems identified with the existing home monitoring system and proposed requirements for the COVID-19 Symptom Monitoring System (CoSMoS).

Problems with the existing home monitoring system	Requirements for CoSMoS
High workload for the district health officials in calling the patients to assess their daily symptoms	A daily automated messaging and reminder system sends active reminders for patients to key in their symptoms every day
Issues of surveillance using phone calls (ie, patients did not pick up the calls, patients worried about scam calls, and long phone conversations to assess each symptom)	Development of a simple system that is easy to use, secure, interactive, and trustworthy
Lack of coordinating care and continuity of care for the patients under investigation and close contacts, especially coordinating care between COVID-19 centers and district health officials	Development of a system to monitor patients with continuity of care
Unnecessary clinic or emergency department visits due to change of symptoms or administrative issues (ie, letter of quarantine) expose the patients and health care workers (HCWs) to COVID-19 infection risk	An automated system performs patients’ risk assessments safely and guides patients in clinical decision making, whether to continue home monitoring or to seek medical attention; a dashboard system provides active monitoring and facilitates the HCW in identifying patients who require teleconsultation and unreported patients

**Figure 1:**
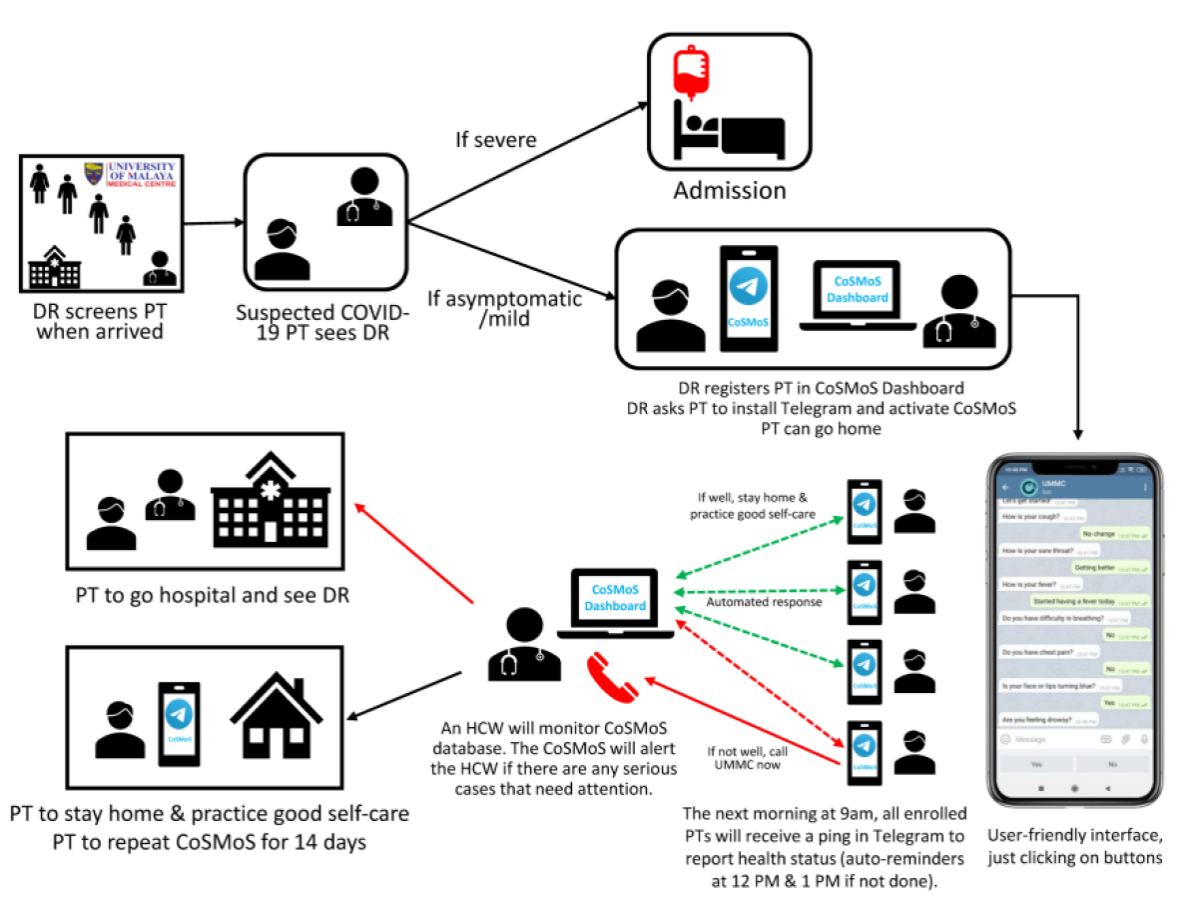
COVID-19 Symptom Monitoring System (CoSMoS) storyboard. DR: doctor; HCW: health care worker; PT: patient; UMMC: University of Malaya Medical Centre.

Besides the three main functionalities, the additional requirements proposed included (1) materials to be sent to patients (ie, privacy policy, user guide, and educational materials), (2) additional notes in the dashboard so that CoSMoS can be used independently without relying on electronic medical records, and (3) a data export function in the dashboard for external data analysis. A storyboard on how CoSMoS will be used from enrollment to completion of monitoring was used to illustrate the system more clearly and thoroughly (see Figure 1).

#### Algorithm and Content Drafting

A total of 13 iterative cycles, including major and minor changes, were done to refine the clinical algorithm (see Multimedia Appendix 2). The text for questions and answer options were refined so that they were easily understandable by the layperson. An end message would be sent to patients automatically based on the algorithm to inform patients whether they could stay at home or call the HCW for teleconsultation. A safety net message advised the patients to attend nearby health care facilities if they were unable to reach the HCW for teleconsultation. The algorithm had a lower threshold to prompt a patient to call the HCW because patient safety was taken into significant consideration. A free-text remark field was created as another safety net measure for the patients. The algorithm was translated into Malay and Chinese languages to suit local needs.

### Phase 2: Development Testing Iteration

#### Development Platform

The technical team first proposed to employ WhatsApp as the platform to deliver the symptom assessment function, as WhatsApp is widely used in Malaysia. However, the plan was halted, as obtaining the WhatsApp Business application programming interface required a long approval time. The team then proceeded with Telegram, as it could provide the solution without complicated approval.

CoSMoS is hosted on the DigitalOcean cloud infrastructure. GitHub was used for code repository, Trello was used for task management, Slack was used for discussions on technical issues, and WhatsApp was used for communication between core and technical teams. Development tools and languages included the React JavaScript library for the clinician dashboard and Go for the back end. Firebase was first chosen for the database and Algolia for the search engine, but it was switched to MongoDB due to performance and cost considerations. Docker Hub was used for container images, and Sketch software was used to create CoSMoS’s logo. In addition, Freshdesk was used to set up a technical help desk, through which the technical team received issues and reports from the clinical team. Kibana was used for logging server activities and for auditing purposes. The CI/CD pipeline enabled the seamless integration of multiple tools used in the development CoSMoS.

#### System Architecture

**Figure 2:**
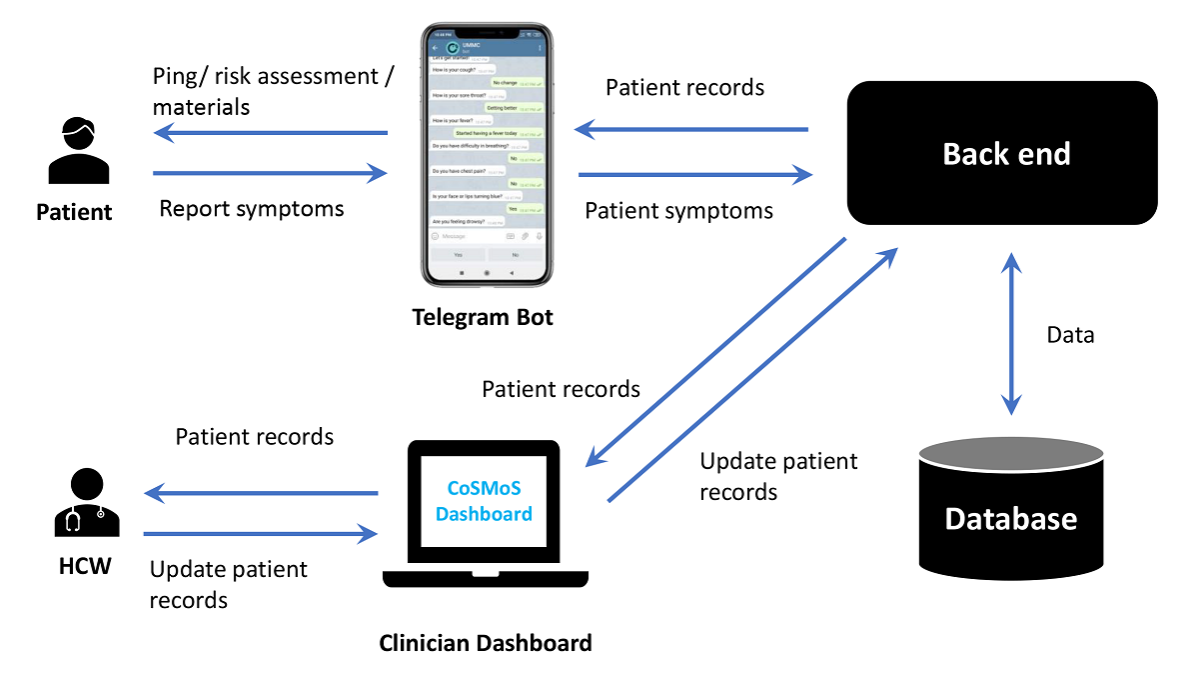
System architecture of the COVID-19 Symptom Monitoring System (CoSMoS). HCW: health care worker.

The architecture of CoSMoS is shown in Figure 2. From the user’s end, the Telegram bot retrieves PUIs’ records from the database through the back end. Ping messages are sent to the PUIs at 9 AM, 12 PM, and 1 PM daily to remind the patients to report their symptoms. The PUIs use the Telegram bot to report their daily symptoms. The symptoms are sent to the back end before being stored in the database; the HCWs monitor PUIs’ symptoms through the clinician dashboard at 2 PM every day. PUIs’ daily statuses are grouped into four main categories:

1. Category A. Reported: unstable and called the HCW.
2. Category B. Reported: unstable, but yet to call the HCW.
3. Category C. Reported: stable.
4. Category D. Not reported.

For category A, the patient would be assessed immediately over the phone (ie, teleconsultation) as they called the HCW. For categories B and D, the HCW would contact patients at 2 PM during the clinical review. There is no need to contact patients for category C unless the HCW identified serious comments in the open-ended remark box during the review. The HCWs may update the PUIs’ records, such as changing a PUI’s status from *asymptomatic* to *symptomatic* or *completed monitoring*, entering a PUI’s swab test result, and adding a note to a PUI’s record. The HCWs can also export PUIs’ records in comma-separated vector format. Retrieving, updating, and storing PUIs’ records between the dashboard and database would also be done through the back end, preserving data security and integrity with mechanisms such as authentication, authorization, and validation.

#### Iterative Development Testing Cycles

There were many cycles of specify-develop-test-revise of CoSMoS, as this study used the agile development method. The tester team, which was independent from the developers, documented test cases that were used and reused to ensure quality and efficiency of testing; see example test cases in Multimedia Appendix 3. The core team continuously gave feedback to the technical team after testing new deployments to the staging environment daily. However, there were three main prototype checkpoints—CoSMoS prototype 1, CoSMoS prototype 2, and finalized CoSMoS—to present major changes made to the system. Thus, it can be considered that the development process comprised three major agile cycles.

The clinicians were the main help desk contact people when patients had any technical issues. Patients could call the hotline to the clinician directly for technical issues. If the clinicians could not solve an issue, the technical issue would be channeled to the technical help desk system.

### Phase 3: Integration Into Clinical Practice

Textbox 1 shows the technical and clinical implementation barriers identified during the simulation and role-playing. The workflow was improved by introducing new strategies, such as refining the patients’ recruitment criteria for CoSMoS and creating a communication script for HCWs to explain CoSMoS to patients. We also prepared a patient’s and doctor’s guide to CoSMoS and Telegram bot installation.

**Technical and clinical implementation barriers of the COVID-19 Symptom Monitoring System (CoSMoS).**

**Technical barriers:**

- Poor internet connectivity
- Insufficient digital storage to download the Telegram app by suspected COVID-19 patients
- Inadequate mobile data quota to download an app

**Clinical implementation barriers:**

- Ineffective communication between health care workers (HCWs) and patients (ie, enrollment in CoSMoS)
- CoSMoS prolonged the consultation time (ie, longer contact time between HCWs and suspected COVID-19 patients) for enrollment and the installation of CoSMoS
- Patient factors (ie, unfamiliarity with digital technology)

**Figure 3:**
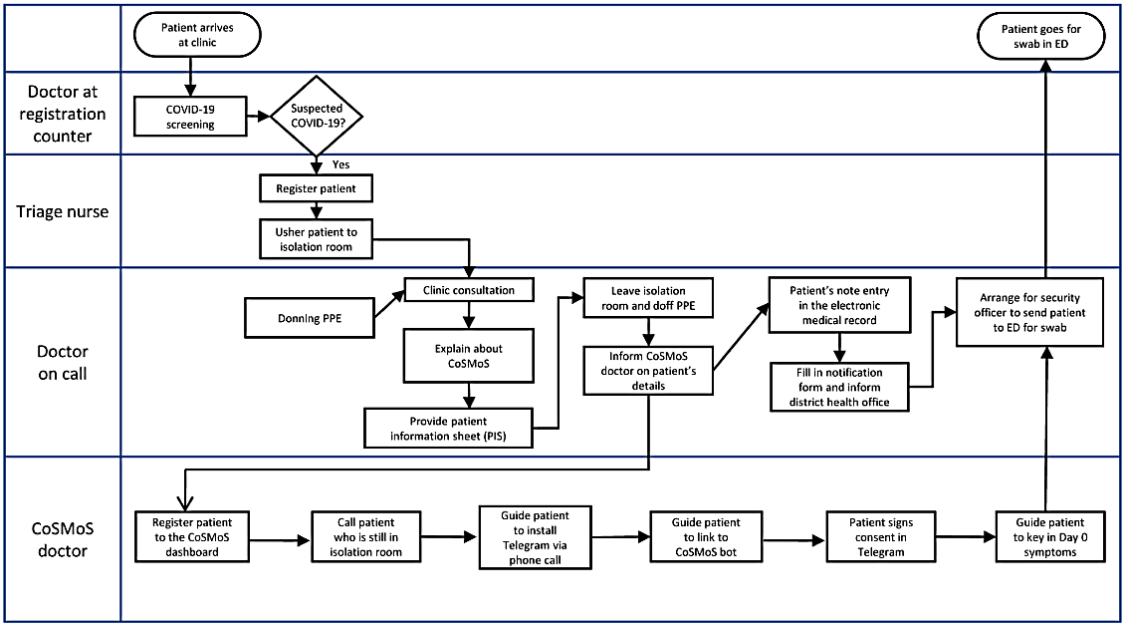
Integration of the COVID-19 Symptom Monitoring System (CoSMoS) into the clinical setting. ED: emergency department; PPE: personal protective equipment.

Because of the high infectivity of COVID-19, necessary precautions were taken to ensure the safety of both patients and HCWs. Patient enrollment procedures, informed consent–taking, and CoSMoS installation were done remotely by a CoSMoS doctor via phone call to reduce the physical contact time. Figure 3 shows the clinical workflow of integrating CoSMoS into clinical practice. The teleconsultation of CoSMoS was done following the Malaysian Medical Council advisory on virtual consultation [12]. The CoSMoS app served as a complementary service to the existing manual home monitoring system by the public health offices, not as a replacement, to ensure that the safety of patients was not compromised.

## Discussion

### Principal Findings

This paper presents the agile development process of a digital health innovation during the COVID-19 pandemic within a short period. CoSMoS was the first automated monitoring system aimed at the Malaysian population to provide home monitoring and phone consultation for patients with suspected COVID-19. There was an urgent need for CoSMoS to reduce the burden on the health care system while providing a better home monitoring service to patients in Malaysia. This paper reports on a systematic approach to its development involving clinical experts, evidence-based clinical guidelines, data security and privacy considerations, and real-life clinical integration of an eHealth app during a pandemic.

Several COVID-19 symptom monitoring systems were developed and deployed in developed countries, such as France, China, and Korea [6,13,14]. Covidom, a web-based application developed in France, was used on a large scale for home monitoring of patients with COVID-19 [6]. A similar approach was used in China, where the telemedicine system was developed based on a popular social media platform, WeChat, to monitor those who needed to be quarantined at home [13]. In this study, the CoSMoS symptom monitoring bot was delivered via the Telegram app; this app is commonly used among Malaysians, as government ministries and agencies disseminate COVID-19 news and health awareness via Telegram. Using the existing digital platform would be more user friendly for patients as they are familiar with the app. In the Korean military hospitals, a similar COVID-19 symptom monitoring app was developed with an additional model prediction programming interface to predict whether a patient requires attention from health care professionals [14]. This function was not developed in CoSMoS because of limited time, data, and resources required to accurately predict patient outcomes. The automated CoSMoS was useful in alleviating the burden on the health care system, reducing physical contact, and reducing the risk of delayed hospitalization [6,13]. The detailed developmental process described in this paper could be transposed to other developing countries where resources are limited to engaging commercial developers. Using existing local expertise and available resources, developing a digital solution for a COVID-19 symptom monitoring system is feasible and applicable for urgent use in the local setting.

The time factor was the most critical challenge to the development of CoSMoS because an automated COVID-19 home monitoring system was urgently needed in the health care system to combat the COVID-19 pandemic. Compared to the usual developmental process of a monitoring system for chronic diseases [12,15,16], conducting a detailed patient needs assessment was not feasible during the COVID-19 pandemic, due to Malaysia’s movement control restriction situation. Hence, the initial requirement formation from Phase 1 was done with input from clinical experts who would be the users for the system and with a review of the existing guidelines for COVID-19 home monitoring. We were able to develop CoSMoS in 19 days because many experienced developers were organized into specialized teams according to their expertise (ie, front end, back end, Telegram bot, and infrastructure) and were led by computer science academicians who worked long hours. Each development task was systematically delegated to the designated team using a task management tool (ie, Trello). Each task was granular enough to be executed by a team member for accountability. Progression of the task to completion was also monitored using Trello. Thus, the development project was adequately managed amid changing requirements. Using an existing app, such as the Telegram app that was used in this study, instead of developing a new bot system reduced the development time. CoSMoS is an independent system that does not require integration into the existing electronic medical record system; its development and deployment into clinical practice were simpler.

This study recognized that the successful development of CoSMoS required effective communication between the technical and clinical teams. Communication and cooperation challenges are common in the development process of an eHealth project, especially where experts from different working cultures and backgrounds are involved [17]. In this CoSMoS project, communication was conducted via virtual meetings (ie, Zoom) and electronic platforms, such as chat groups and emails, due to the movement control restriction in Malaysia. However, communication was effective as both clinical and technical teams understood each other’s languages and terminologies, work processes, work practices, and limitations as the team collaborated for a few years. The team leaders and patient advocate in the development team also played a prominent role in coordinating the collaboration. The change requests for CoSMoS were made via virtual meetings during the initial development phase using the agile development method. The communication method had changed from virtual meetings to the help desk method after CoSMoS was developed to enable better management and documentation of system evolution.

The consequential feature of CoSMoS development was the concept of combining a home monitoring app and teleconsultation with doctors. Consideration of patient safety was of the utmost importance, especially in designing the decision-making clinical algorithm and the active telemonitoring system that would help patients be safely monitored at home [18]. There were several challenges in the integration of this interactive and real-time patient monitoring system in clinical practice. Given the high infectivity of COVID-19, research and clinical ethics were considered in the clinical workflow process. The enrollment process was done via phone call to reduce patients’ and doctors’ exposure times in order to prevent transmission of COVID-19 infection to the health care providers. Several rounds of role-playing between the CoSMoS core members and the clinic staff provided useful feedback and change requests to improve the CoSMoS app and dashboard. The role-playing managed to streamline the integration process, identify technical problems, and reduce the risks. To ensure patients’ safety, the clinical workflow of CoSMoS was parallel to the usual care provided by the district health officer so that the care of patients would not be compromised with the introduction of new technology. Patient data privacy and confidentiality is often a main concern, especially during a pandemic [19]. There was social stigma toward patients with confirmed COVID-19 infection [20]. Privacy and data protection were ensured in CoSMoS by applying authentication and authorization mechanisms. Thus, only authorized personnel were granted access to the data according to their rights of access. Each of the health care providers had their own access identity and passwords to access the CoSMoS dashboard. The data from CoSMoS were not shared with other parties or service settings. Data were transmitted over the internet using https secure communication protocol, Telegram messages were encrypted end-to-end, and the database was hosted on a trusted cloud infrastructure provider (ie, DigitalOcean) with clearly defined privacy and security policies. Internally, the technical team had also set a security policy to control access to the patient database.

### Strengths

Strengths of this paper include a full description of the CoSMoS development process, which could be replicated for future health system development during a pandemic. We used a patient-centered care approach in the development process to consider the system’s integration into clinical practice. The development process of CoSMoS prioritized the health and safety of patients and health care providers.

### Limitations

A limitation of this development process was that usability testing was only done among computer science students and health care providers. It was a clinical challenge to test the system on real patients given the infectivity of COVID-19 and considering researchers’ safety. The usability testing reported in this paper did not represent the target population. The usability and utility evaluation via a qualitative study was conducted separately.

### Conclusions

In view of the urgent need of a COVID-19 symptom monitoring system during the COVID-19 pandemic, we have shown that one can be developed systematically and practically within a short period using the agile model. With effective communication between the technical and clinical teams, developing a digital health care monitoring system is feasible and practicable without compromising patient safety. The development process described in this paper and the lessons learned could guide the development of a digital monitoring system during the next pandemic.

## Appendix

Multimedia Appendix 1 Development timeline of the COVID-19 Symptom Monitoring System (CoSMoS).

Multimedia Appendix 2 Clinical algorithm.

Multimedia Appendix 3 COVID-19 Symptom Monitoring System (CoSMoS) test cases.

## Abbreviations

CI/CD: continuous integration and continuous deployment
CoSMoS: COVID-19 Symptom Monitoring System
HCW: health care worker
PUI: patient under investigation
UMMC: University of Malaya Medical Centre

## References

[1] Zastrow M (2020). Coronavirus contact-tracing apps: Can they slow the spread of COVID-19?. Nature.

[2] Gong M, Liu L, Sun X, Yang Y, Wang S, Zhu H (2020). Cloud-based system for effective surveillance and control of COVID-19: Useful experiences from Hubei, China. J Med Internet Res.

[3] Ting DSW, Carin L, Dzau V, Wong TY (2020). Digital technology and COVID-19. Nat Med.

[4] Editorial (2020). Show evidence that apps for COVID-19 contact-tracing are secure and effective. Nature.

[5] Ienca M, Vayena E (2020). On the responsible use of digital data to tackle the COVID-19 pandemic. Nat Med.

[6] Yordanov Y, Dechartres A, Lescure X, Apra C, Villie P, Marchand-Arvier J, Debuc E, Dinh A, Jourdain P, AP-HP / Universities / Inserm COVID-19 Research Collaboration (2020). Covidom, a telesurveillance solution for home monitoring patients with COVID-19. J Med Internet Res.

[7] (2020). Considerations for quarantine of contacts of COVID-19 cases. World Health Organization.

[8] (2020). COVID-19 management guidelines in Malaysia. Ministry of Health Malaysia.

[9] Lalband N, Kavitha D (2019). Software engineering for smart healthcare applications. Int J Innov Technol Explor Eng.

[10] Matthew-Maich N, Harris L, Ploeg J, Markle-Reid M, Valaitis R, Ibrahim S, Gafni A, Isaacs S (2016). Designing, implementing, and evaluating mobile health technologies for managing chronic conditions in older adults: A scoping review. JMIR Mhealth Uhealth.

[11] Song Y, Lu Y (2015). Decision tree methods: Applications for classification and prediction. Shanghai Arch Psychiatry.

[12] Adu MD, Malabu UH, Malau-Aduli AEO, Malau-Aduli BS (2020). The development of My Care Hub mobile-phone app to support self-management in Australians with type 1 or type 2 diabetes. Sci Rep.

[13] Xu H, Huang S, Qiu C, Liu S, Deng J, Jiao B, Tan X, Ai L, Xiao Y, Belliato M, Yan L (2020). Monitoring and management of home-quarantined patients with COVID-19 using a WeChat-based telemedicine system: Retrospective cohort study. J Med Internet Res.

[14] Heo J, Park JA, Han D, Kim H, Ahn D, Ha B, Seog W, Park YR (2020). COVID-19 outcome prediction and monitoring solution for military hospitals in South Korea: Development and evaluation of an application. J Med Internet Res.

[15] Sadasivam RS, Delaughter K, Crenshaw K, Sobko HJ, Williams JH, Coley HL, Ray MN, Ford DE, Allison JJ, Houston TK (2011). Development of an interactive, web-delivered system to increase provider-patient engagement in smoking cessation. J Med Internet Res.

[16] Nota I, Drossaert CHC, Melissant HC, Taal E, Vonkeman HE, Haagsma CJ, van de Laar MAFJ (2017). Development of a web-based patient decision aid for initiating disease modifying anti-rheumatic drugs using user-centred design methods. BMC Med Inform Decis Mak.

[17] Petersen LS, Bertelsen P, Bjørnes C (2013). Cooperation and communication challenges in small-scale eHealth development projects. Int J Med Inform.

[18] Parimbelli E, Bottalico B, Losiouk E, Tomasi M, Santosuosso A, Lanzola G, Quaglini S, Bellazzi R (2018). Trusting telemedicine: A discussion on risks, safety, legal implications and liability of involved stakeholders. Int J Med Inform.

[19] Li J, Seale H, Ray P, Rawlinson W, Lewis L, Macintyre CR (2012). Issues regarding the implementation of eHealth: Preparing for future influenza pandemics. Interact J Med Res.

[20] Singh R, Subedi M (2020). COVID-19 and stigma: Social discrimination towards frontline healthcare providers and COVID-19 recovered patients in Nepal. Asian J Psychiatr.

