# Supplementary figures and images for: An Automated Patient Self-Monitoring System to Reduce Health Care System Burden During the COVID-19 Pandemic in Malaysia: Development and Implementation Study
Source: JMIR Med Inform. 2021 Feb 26;9(2):e23427. doi: 10.2196/23427 (PMC7919845; doi:10.2196/23427)

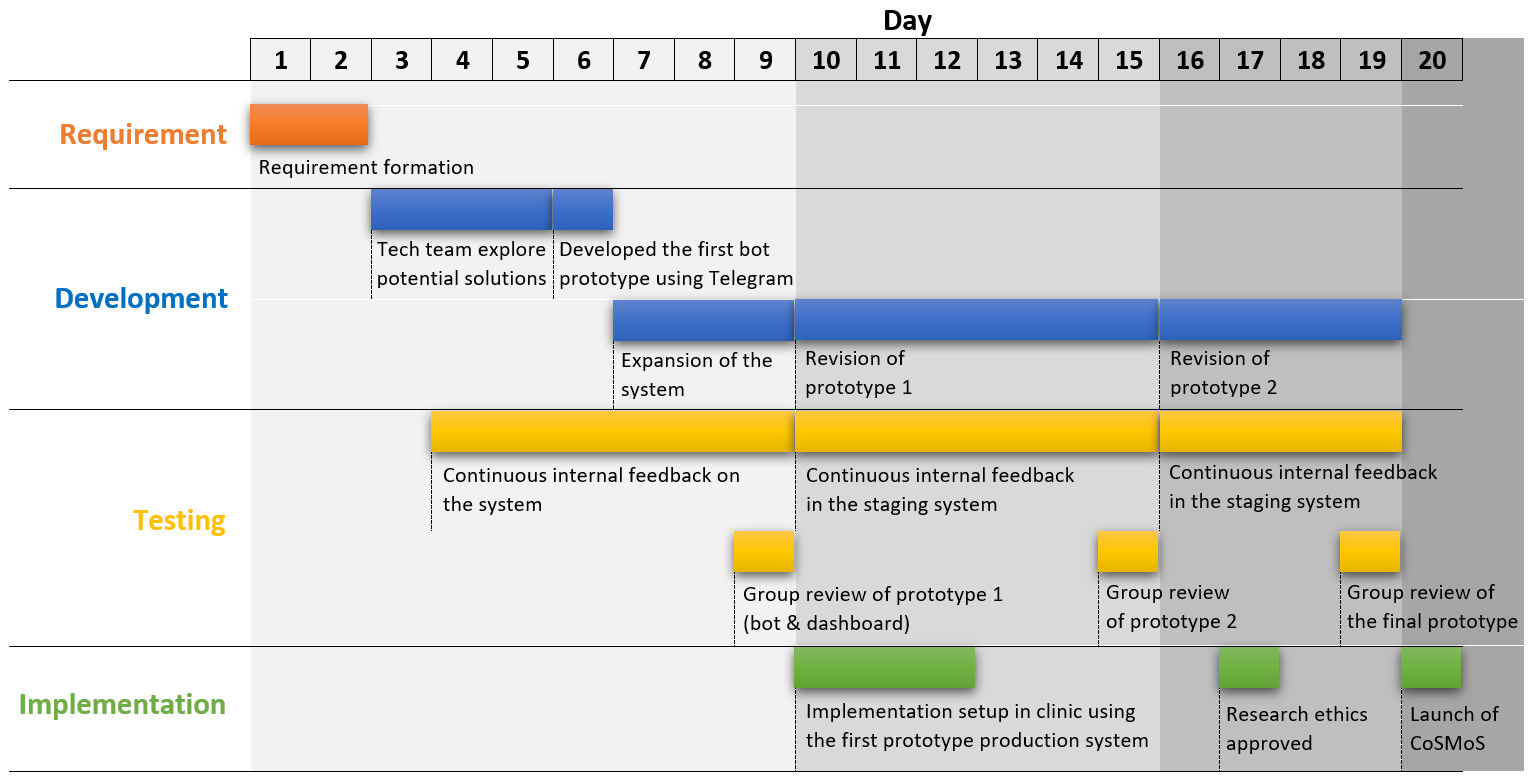

Supplement: Multimedia Appendix 1 [file medinform_v9i2e23427_app1.png]
