# Supplementary material for: An Automated Patient Self-Monitoring System to Reduce Health Care System Burden During the COVID-19 Pandemic in Malaysia: Development and Implementation Study
Source: JMIR Med Inform. 2021 Feb 26;9(2):e23427. doi: 10.2196/23427 (PMC7919845; doi:10.2196/23427)
Supplement: Multimedia Appendix 2 [file medinform_v9i2e23427_app2.pdf]

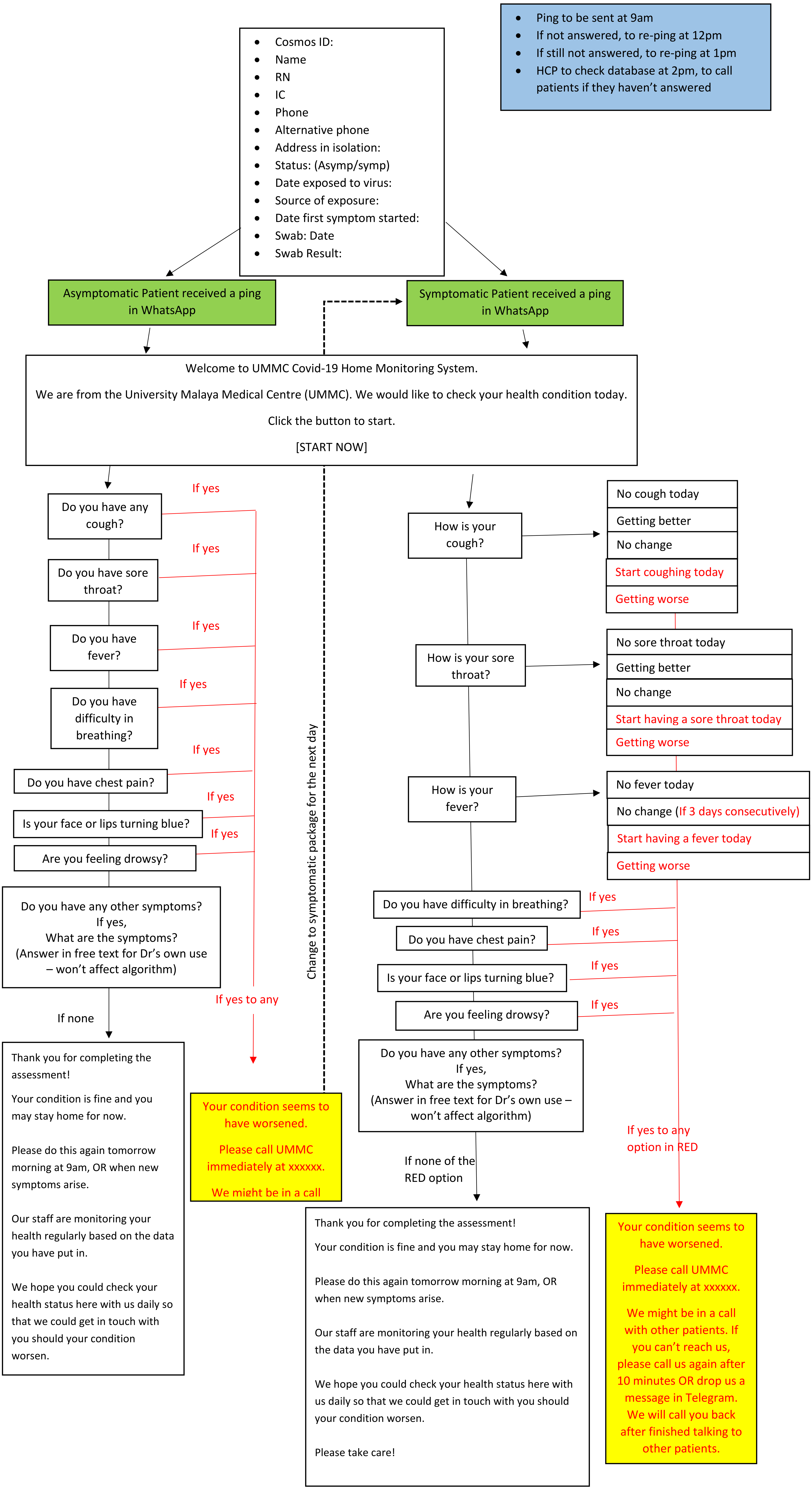
Ping to be sent at 9am
If not answered, to re-ping at 12pm
If still not answered, to re-ping at 1pm
HCP to check database at 2pm, to call patients if they haven't answered
Cosmos ID:
Name
RN
IC
Phone
Alternative phone
Address in isolation:
Status: (Asymp/symp)
Date exposed to virus:
Source of exposure:
Date first symptom started:
Swab: Date
Swab Result:
Asymptomatic Patient received a ping in WhatsApp
Symptomatic Patient received a ping in WhatsApp
Welcome to UMMC Covid-19 Home Monitoring System.
We are from the University Malaya Medical Centre (UMMC). We would like to check your health condition today.
Click the button to start.
[START NOW]
Do you have any cough?
If yes
Do you have sore throat?
If yes
Do you have fever?
If yes
Do you have difficulty in breathing?
If yes
Do you have chest pain?
If yes
Is your face or lips turning blue?
If yes
Are you feeling drowsy?
If yes
Do you have any other symptoms? If yes, What are the symptoms? (Answer in free text for Dr's own use – won't affect algorithm)
If none
If yes to any
Thank you for completing the assessment!
Your condition is fine and you may stay home for now.
Please do this again tomorrow morning at 9am, OR when new symptoms arise.
Our staff are monitoring your health regularly based on the data you have put in.
We hope you could check your health status here with us daily so that we could get in touch with you should your condition worsen.
Your condition seems to have worsened.
Please call UMMC immediately at xxxxxx.
We might be in a call
Change to symptomatic package for the next day
How is your cough?
No cough today
Getting better
No change
Start coughing today
Getting worse
How is your sore throat?
No sore throat today
Getting better
No change
Start having a sore throat today
Getting worse
How is your fever?
No fever today
No change (If 3 days consecutively)
Start having a fever today
Getting worse
Do you have difficulty in breathing?
If yes
Do you have chest pain?
If yes
Is your face or lips turning blue?
If yes
Are you feeling drowsy?
If yes
Do you have any other symptoms? If yes, What are the symptoms? (Answer in free text for Dr's own use – won't affect algorithm)
If none of the RED option
If yes to any option in RED
Thank you for completing the assessment!
Your condition is fine and you may stay home for now.
Please do this again tomorrow morning at 9am, OR when new symptoms arise.
Our staff are monitoring your health regularly based on the data you have put in.
We hope you could check your health status here with us daily so that we could get in touch with you should your condition worsen.
Please take care!
Your condition seems to have worsened.
Please call UMMC immediately at xxxxxx.
We might be in a call with other patients. If you can't reach us, please call us again after 10 minutes OR drop us a message in Telegram. We will call you back after finished talking to other patients.
