# Supplementary material for: An Automated Patient Self-Monitoring System to Reduce Health Care System Burden During the COVID-19 Pandemic in Malaysia: Development and Implementation Study
Source: JMIR Med Inform. 2021 Feb 26;9(2):e23427. doi: 10.2196/23427 (PMC7919845; doi:10.2196/23427)
Supplement: Multimedia Appendix 3 [file medinform_v9i2e23427_app3.pdf]

# CoSMoS Test Cases

## Table of Contents

**Test Template** 4
Scenario : User adds new patient with correct information 4

1.0 Admin Dashboard 5
1.1 Old Patients 5
1.1.1 Feature : Taking health assessment 5
1.1.1.1 Scenario : Old user tries to access health assessment again. 5
1.2 International Patients 6
1.2.1 Feature : Registering new patients 6
1.2.1.1 Scenario : User tries to add new international patient (eg: China) 6
1.3 “Today” Labelling 7
1.3.1 Feature : New reports today will be labelled 8
1.3.1.1 Scenario : User takes health assessment 8
1.4 Patient with invalid details 8
1.4.1 Registering new patient 9
1.4.1.1 Scenario: User tries to add new local patient 9
1.4.1.2 Scenario: Patient with 10
1.5 User Table 11
1.5.1 Feature : User Search 11
1.5.1.1 Scenario : User search by name, phonenum, email 11
1.5.1.2 Scenario : Search details on user lists 11
1.6 Patient with duplicate details (e.g. phone number) 12
1.6.1 Patient with the phone number reports himself/herself 12
1.6.1.1 Scenario: Patient of duplicate phone number registers and reports himself/herself (Has been fixed. Tested by Si Tian) 12
1.7 Export 13
1.7.1 Feature : Exported file details 13
1.7.1.1 Scenario : User exports the swabs table (can be edited well manually in excel) 13
1.8 Invalid Phone Number 14
1.8.1 Feature : Enter an invalid phone number 14
1.8.1.1 Scenario : User enters an invalid phone number 14
1.9 Update Patient Detail 14
1.9.1 Feature : Edit Patient Detail 15
1.9.1.1 Scenario : User try to edit patient’s detail 15
1.10 Reports Table 16
1.10.1 Feature : Update Reports Table 16
1.10.1.1 Scenario : Health assessment is done twice in a day 16
1.10.1.2 Scenario : Doctor remarks has been added 16
1.11 Non-Reported Table 16

1.11.1 Feature : Update Non-Reported Table 17
1.11.1.1 Scenario : Update everyday at 12.00am 17
1.12 Patient Table 18
1.12.1 Feature : Only One Phone Number Allowed 18
1.12.1.1 Scenario : Create a patient with existing phone number 18
1.12.2 Feature : Preferred Language 18
1.12.2.1 Scenario : Admin changes preferred language after the patient has finished a health assessment 18
1.12.3 Feature : Patient status sorting 19
1.12.3.1 Scenario : Admin sorts the status of patient 19
1.12.4 Feature : Create Patient with different preferred language 20
1.12.4.1 Scenario : Create Patient with different preferred language 20
1.12.3 Feature : Adding new patient 21
1.12.3.1 Scenario : Insert patient name with various 'special' Unicode characters 21
1.12.3 Feature : Bot verdict, dashboard symptoms and days since fever from Asymptomatic Patient 24
1.12.3.1 Scenario : Asymptomatic patient takes health assessment with only one “Yes” 24
1.12.3.1 Scenario : Asymptomatic patient takes health assessment with two “Yes” 26
1.13 Dashboard Line Graph 34
1.13.1 Feature : Details 34
1.13.1.1 Scenario : Line graph title 34
1.14 Fever Cont Days 34
1.14.1 Feature : Days with fever 35
1.14.1.1 Scenario : First day of getting fever 35
2.0 Telegram Bot 36
2.1 Health Assessment 36
2.1.1 Feature : Taking health assessment 36
2.1.1.1 Scenario : User uses two test bots 36
2.1.1.3 Scenario : Patient responds with a sticker/GIF (Bug has been fixed) 38
2.1.1.4 Scenario : Accessing bot using unregistered number 38
2.1.1.5 Scenario : Accessing additional information for guidance during Q&A session 39
2.1.1.6 Scenario : Bot in a group 40
2.1.1.7 Scenario : Accessing personal information 41
2.1.1.8 Scenario : User cleared/deleted his history chat 42
2.1.1.9 Scenario: Spamming on the bot with “No” 42
2.1.1.10 Scenario : User accidentally choose the wrong answer on one question 43
2.1.1.11 Scenario :Other symptoms data collection 44
2.1.1.12 Scenario : User responds by forwarding messages 45
2.1.1.13 Scenario : User answered a Mandarin question using English(Yes/No) 46
2.1.1.14 Scenario : User maliciously tries to link other phone numbers 48
2.1.1.15 Scenario : Patients deleted Telegram chats. 49

2.1.1.16 Scenario : Patient clicks on “Yes” and “No” button at the same time 50
2.1.1.17 Scenario : User answers the questions in a different language 50
2.2 Notifications of Health Assessment 52
2.2.1 Feature : Receive Notification Every Morning 52
2.2.1.1 Scenario : Taking Health Assessment Everyday 52
2.2.1.2 Scenario : Late Notification For Taking Health Assessment 53
2.2.2 Feature : Receive Notification Of Prefered Language to Conduct Test 53
2.2.1.1 Scenario : Getting notification of preferred language when assessment for the day isn't done 53

# TODO: Define features and flows

# Test Template

**Feature** : [Main feature]

**Scenario** : [Scenario]
**Given** [precondition]
**And** [other preconditions]
**When** [user action]
**And** [other actions]
**Then** [result]
**And** [other results]

Example:

## **Feature** : Add new patient

### **Scenario** : User adds new patient with correct information

**Given** some precondition
**And** some other precondition
**When** some action by the actor
**And** some other action
**And** yet another action
**Then** some testable outcome is achieved
**And** something else we can check happens too
**But** something else that shouldn't happen

Remarks:
Inputs:
ID: ...
Name: ...
PhoneNumber: ...

Tested by:

# 1.0 Admin Dashboard

## 1.1 Old Patients

### 1.1.1 Feature : Taking health assessment

#### 1.1.1.1 Scenario : Old user tries to access health assessment again.

**Given** the patient has completed the assessment before.
**And** the patient is deleted from the dashboard.
**And** the patient has deleted the UMMC_Bot old chats from Telegram.
**When** the patient searches UMMC_bot from Telegram again
**Then** the bot displays a “Restart” button.
**When** the user presses “Restart”
**Then** the bot shows welcoming text with a “Start” button.
**When** the user presses “Start”
**Then** the bot shows “First, please link your phone number to UMMC database” with a “Link” button.
**When** the user presses “Link” and “Share Contact” buttons.
**Then** the bot shows “This tool is for UMMC patients only. To read….” and a “/Info” button.
**When** the patient presses the “/Info” button.
**Then** the bot shows the pdf file and “/help” button.
**When** the patient presses the “/help” button.
**Then** the bot repeats from the beginning.

**Remarks**:
1. Admin dashboard does not receive any data from deleted patient.
2. Deleted patient cannot access to health assessment.
3. **Result within expectation.**

**Device Used:**
1. Android 8.0
2. Windows 10 (Telegram Web using Google Chrome)

**Suggestion**:
1. For the messages “This tool is for UMMC patients only”, the call-to-action can be clearer. It can include some instructions for non UMMC patients to do.

Tested by: Jia Wei

# 1.2 International Patients

## 1.2.1 Feature : Registering new patients

### 1.2.1.1 Scenario : User tries to add new international patient (eg: China)

**Given** patient is using an international phone number instead of local number
**When** user records name, passport, phone number and status
**And** press "Submit"
**But** gives an error "Invalid phone number."
**When** user re-enter the phone number in a different format
**Then** the system accepts the new patient record.

**Remarks:**
Inputs:
ID: 123123
Name: TestChina
PhoneNumber: 821072814030 (korean number, not china, sorry)

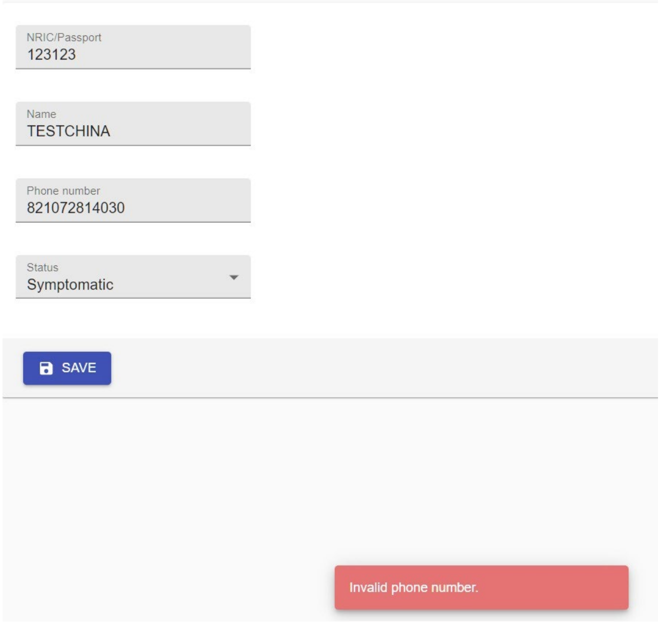


(PS. real number, do not call)
Comments: It seems that the phone number field is very picky on the format of the number.
These work:

1. +82 10-7281-4030
2. +821072814030

This does not work:

1. 821072814030

But oddly enough, these also work:

1. 8612312341234
2. 8613620461140 (real number, don't call.)

**Tested by: Jason and Isaac**

**Tested on:**
Google Chrome version 80.0.3987.163 and Microsoft Edge version 44.18362.449.0

Windows 10 Education build 1909

**Bug fixed and retested on 4/4/2020, now the results are as expected.**

## 1.3 “Today” Labelling

### 1.3.1 Feature : New reports today will be labelled

#### 1.3.1.1 Scenario : User takes health assessment

Given user has been registered as a patient by medical staffs
And user has been notified to take the assessment the next day
And user has done the health assessment
Then the data is found in “Reports” in the admin dashboard
And “Today” is labelled in the correct sequence

Remark: New Feature testing

Tested by: Jia Wei

# 1.4 Patient with invalid details

## 1.4.1 Registering new patient

### 1.4.1.1 Scenario: User tries to add new local patient

**Given** user has access to the dashboard and is trying to help register new patient in the Patient tab
**And** user keys in details of the patient
**Then** user clicks on Save button
**But** "Server unavailable. Please try again" pops up
**And "**Failed to load resource: the server responded with a status of 500 ()" was shown in the console

Remarks:
Expected: Invalid user details errors pop up

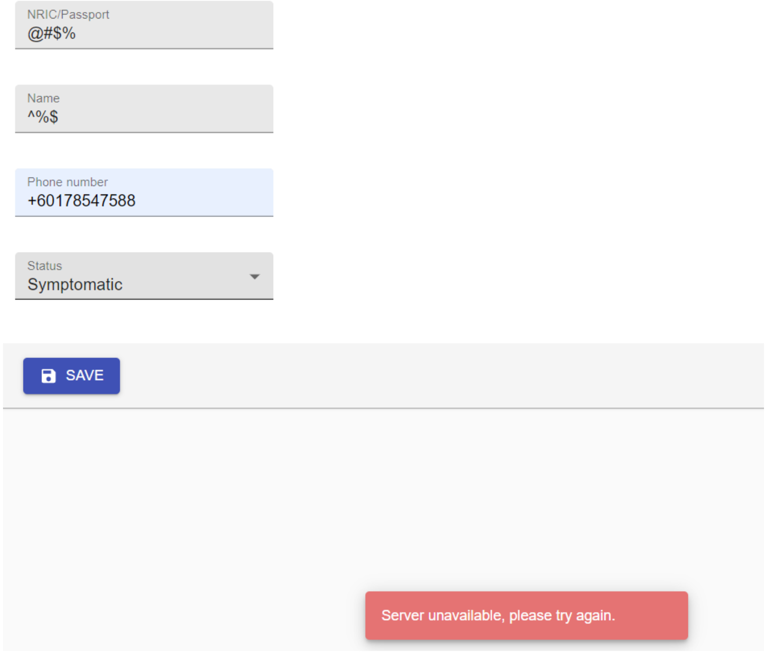


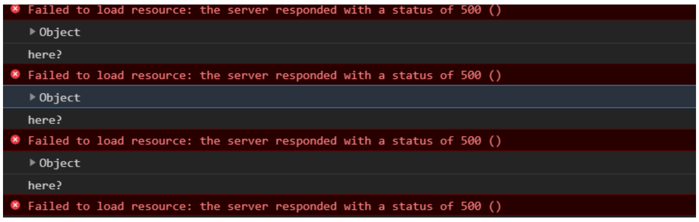


Name: Nat
Phone Number: -

### 1.4.1.2 Scenario: Patient with

**Given** user has access to the dashboard and is trying to help register new patient in the Patient tab
**And** user keys in details of the patient (with local phone number of >13 digits e.g. 0123456789012, 01234567890123, 01234567890124)
**Then** invalid phone number error
**Then** reduce number length to 13 digits (012345678901)
**Then** user clicks on Save button
**Then success**
**But** 012345678901 was displayed as 12345678901

Remarks:
Max for local number is 13 digits
There can be long digits for **NRIC/Passport** (cant be modified later) and **Name** (can be modified later) * why 1 can modify 1 cant
Expected: 012345678901 to be displayed the same

Name: Nat
Phone Number: 012345678901

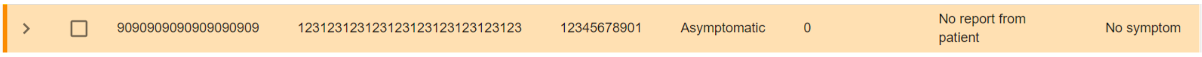

# 1.5 User Table

## 1.5.1 Feature : User Search

### 1.5.1.1 Scenario : User search by name, phonenum, email

Given a list of users in the user table
And user wants to search for another user
And user inserts the name, phonenum, email accordingly/ by each
Then the user finds out the correct user

**Remarks: Results within expectations**

Tested by: Si Tian

### 1.5.1.2 Scenario : Search details on user lists

Given a list of users in the user table
And user wants to search for the user id (disabled columns)
And user can't find as they are hidden.

**Remarks: Results within expectations**

Tested by: Si Tian

# 1.6 Patient with duplicate details (e.g. phone number)

## 1.6.1 Patient with the phone number reports himself/herself

### 1.6.1.1 Scenario: Patient of duplicate phone number registers and reports himself/herself (Has been fixed. Tested by Si Tian)

**Given** 3 users with same phone number registered themselves (2 symptomatic 1 asymptomatic)
**And** user keys in details of the patient
**Then** user clicks on Save button
**But** no duplicate number error popped up when registering patient
**And** identification of user (differentiate users of the same number) before self-testing
**And** only 1 of the numbers was updated

Remarks:
Expected: Duplicate phone number error pops up or identification of user of same phone number before doing Q&A self testing

Name: Nat
Phone Number: 60178547588

Search
0178547588

\+ CREATE ⬇ EXPORT

| | | NRIC/Passport ↑ | Name | Phone number | Status | Days since exposure | Last report | Last report result |
|---|---|---|---|---|---|---|---|---|
| > | ☐ | ASD | ^%$ | 60178547588 | Symptomatic | 0 | 04/04/2020, 16:22:14 TODAY | No symptom |
| > | ☐ | NOTPASSPORT | NATHANIEL2 | 60178547588 | Asymptomatic | 2 | No report from patient | No symptom |
| > | ☐ | NRIC | NATHANIEL | 60178547588 | Symptomatic | 1 | No report from patient | No symptom |

Rows per page: 10 ▾ 1-3 of 3

# 1.7 Export

## 1.7.1 Feature : Exported file details

### 1.7.1.1 Scenario : User exports the swabs table (can be edited well manually in excel)

Given a list of swabs result
And user clicks ‘Export’ to export the tables out for certain purposes
And the excel file coming out
Then all details are demonstrated inside
**But certain details are not clear (such as ID/IC due to the cell formatting)**

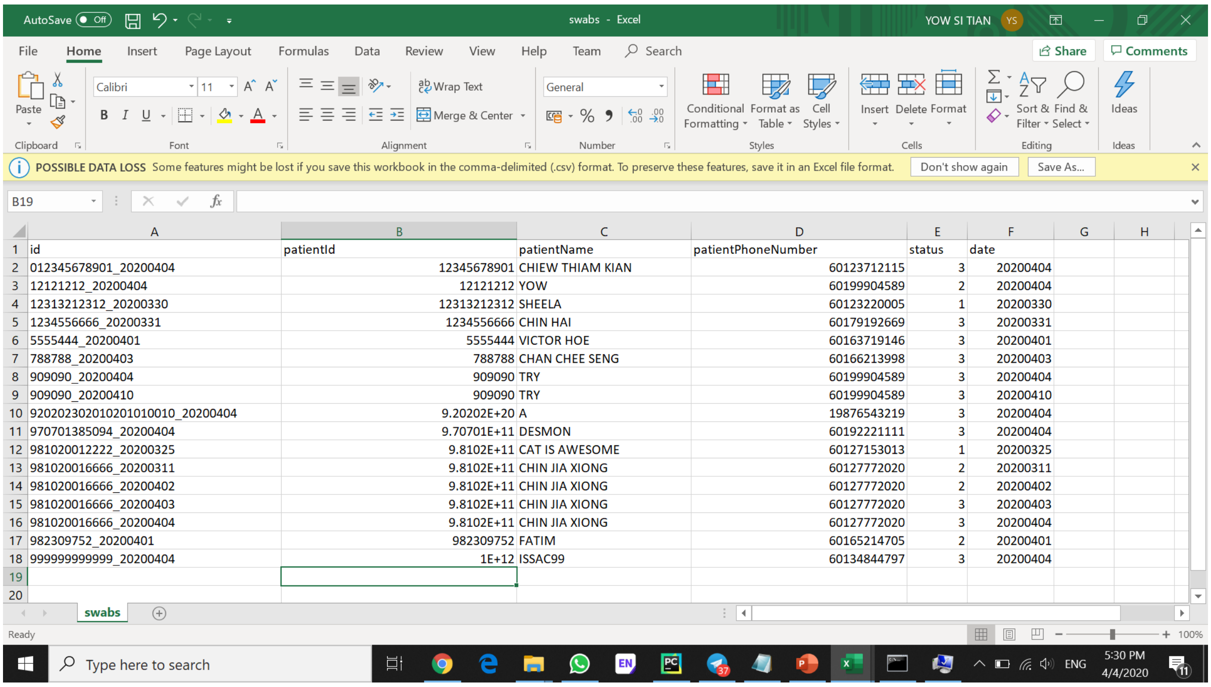

| id | patientId | patientName | patientPhoneNumber | status | date |
|---|---|---|---|---|---|
| 012345678901_20200404 | 12345678901 | CHIEW THIAM KIAN | 60123712115 | 3 | 20200404 |
| 12121212_20200404 | 12121212 | YOW | 60199904589 | 2 | 20200404 |
| 12313212312_20200330 | 12313212312 | SHEELA | 60123220005 | 1 | 20200330 |
| 1234556666_20200331 | 1234556666 | CHIN HAI | 60179192669 | 3 | 20200331 |
| 5555444_20200401 | 5555444 | VICTOR HOE | 60163719146 | 3 | 20200401 |
| 788788_20200403 | 788788 | CHAN CHEE SENG | 60166213998 | 3 | 20200403 |
| 909090_20200404 | 909090 | TRY | 60199904589 | 3 | 20200404 |
| 909090_20200410 | 909090 | TRY | 60199904589 | 3 | 20200410 |
| 92020230201020101010_20200404 | 9.20202E+20 | A | 19876543219 | 3 | 20200404 |
| 970701385094_20200404 | 9.70701E+11 | DESMON | 60192221111 | 3 | 20200404 |
| 981020012222_20200325 | 9.8102E+11 | CAT IS AWESOME | 60127153013 | 1 | 20200325 |
| 981020016666_20200311 | 9.8102E+11 | CHIN JIA XIONG | 60127772020 | 2 | 20200311 |
| 981020016666_20200402 | 9.8102E+11 | CHIN JIA XIONG | 60127772020 | 2 | 20200402 |
| 981020016666_20200403 | 9.8102E+11 | CHIN JIA XIONG | 60127772020 | 3 | 20200403 |
| 981020016666_20200404 | 9.8102E+11 | CHIN JIA XIONG | 60127772020 | 3 | 20200404 |
| 982309752_20200401 | 982309752 | FATIM | 60165214705 | 2 | 20200401 |
| 999999999999_20200404 | 1E+12 | ISSAC99 | 60134844797 | 3 | 20200404 |

**Remarks: for the IC numbers starts from 0, the ‘0’ will be removed automatically.**

**May try to remove the excel formatting or to be easier remove the whole column of patientid since it's similar with the id just that the id has the date behind.**

**When the correct IC number is entered, the resulted IC coming out will be sth like 3.42423E+11**
Tested by: Si Tian
Phone number: 0199904589

# 1.8 Invalid Phone Number

## 1.8.1 Feature : Enter an invalid phone number

### 1.8.1.1 Scenario : User enters an invalid phone number

User enter patient’s details
And user enters an invalid phone number
And the system is suppose to detect it is an invalid phone number
But the system did not pop out an “invalid phone number” notice
And the invalid phone number is successfully entered into the system.

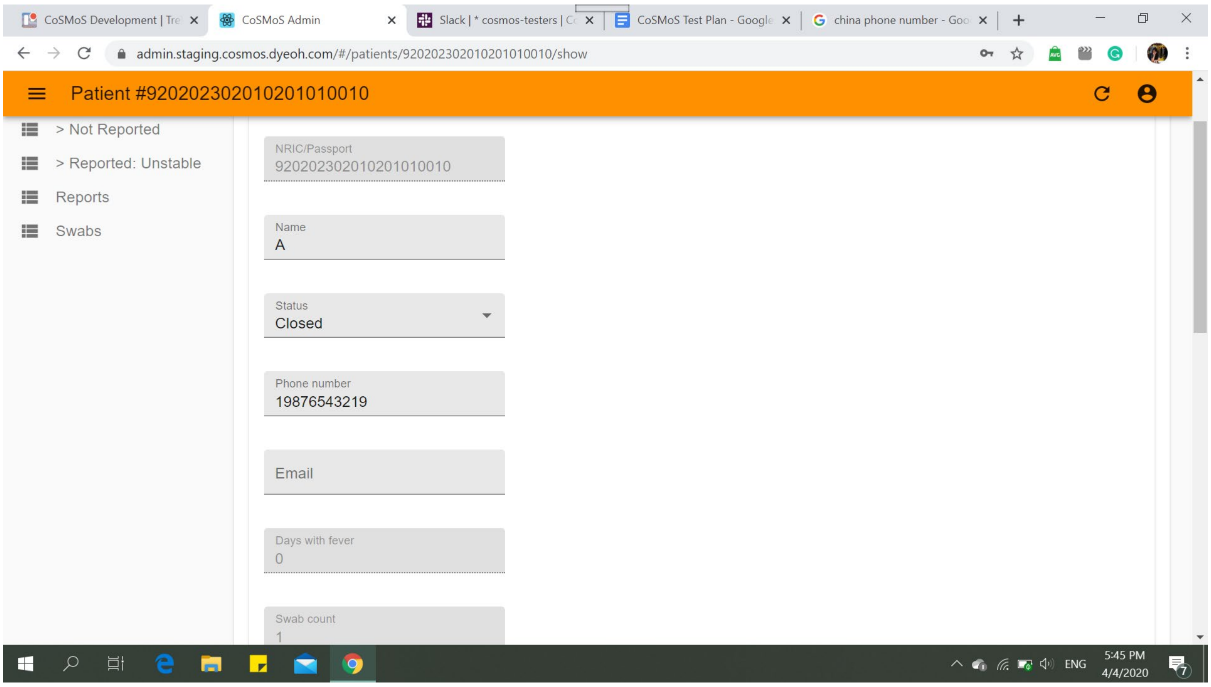


Test by: Huei Ching

# 1.9 Update Patient Detail

## 1.9.1 Feature : Edit Patient Detail

### 1.9.1.1 Scenario : User try to edit patient's detail

User click into an existing patient
And user try to change the name of the patient(maybe due to typo)
But the system did not allow user to do so because of the same phone number(no detail can be updated)

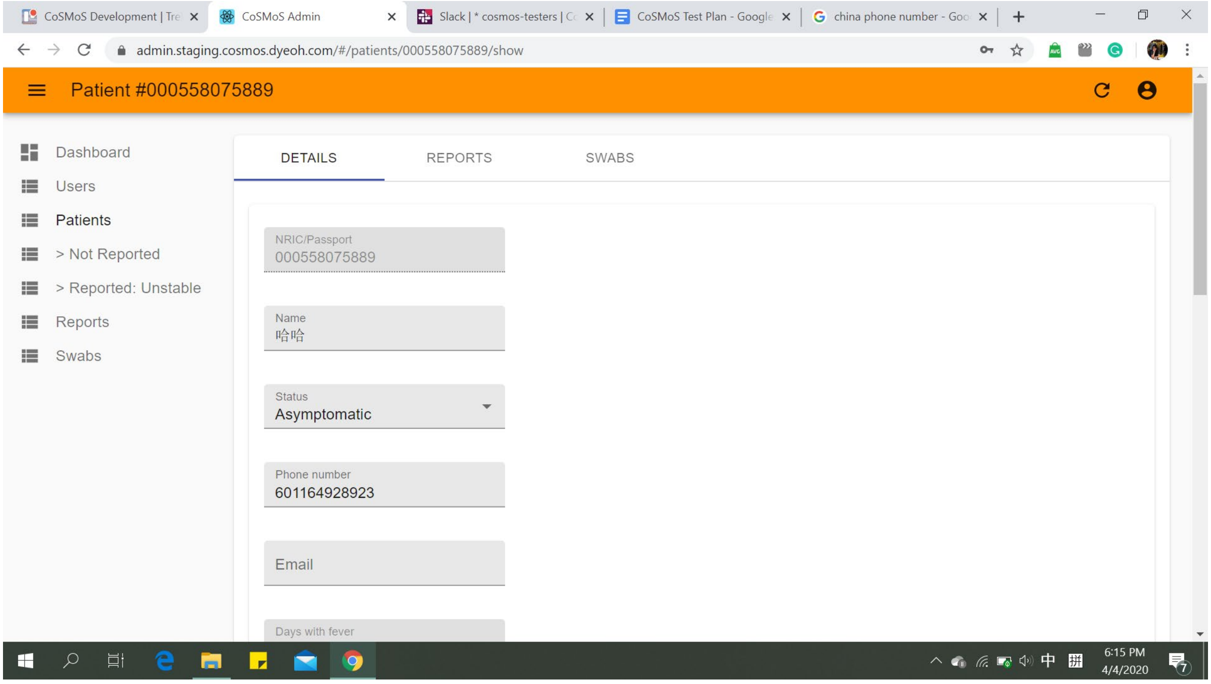


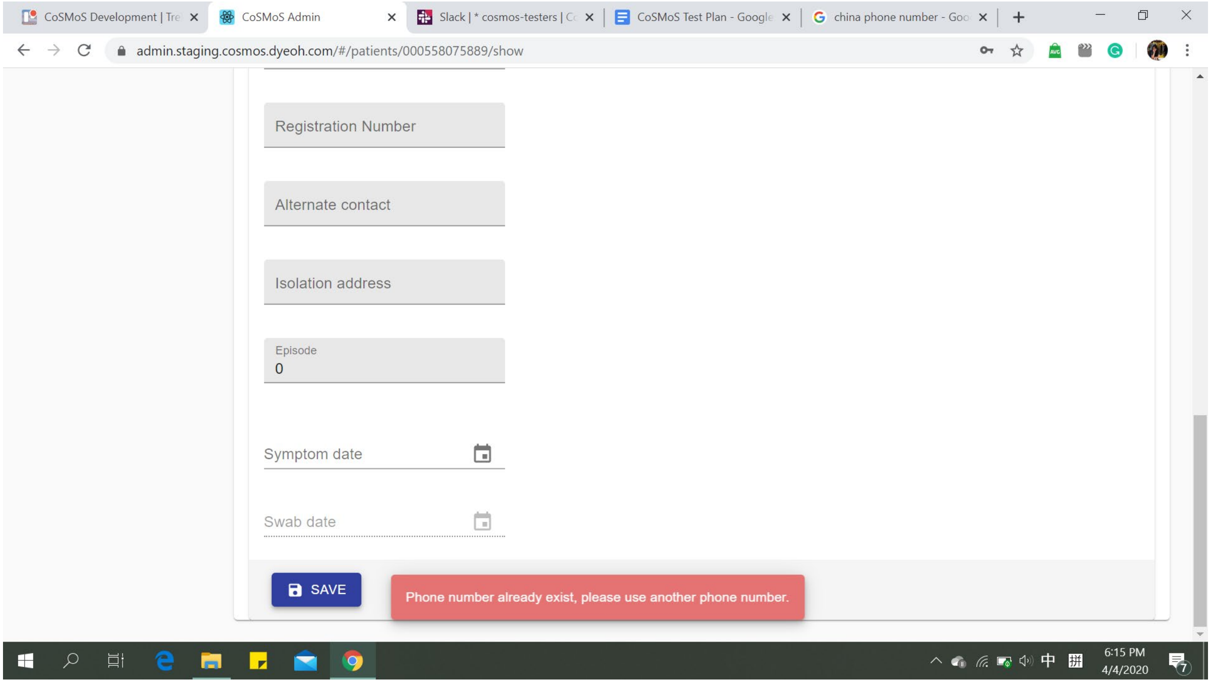


Tested by: Huei Ching

# 1.10 Reports Table

## 1.10.1 Feature : Update Reports Table

### 1.10.1.1 Scenario : Health assessment is done twice in a day

**Given** the user completed the health assessment twice in a day.
**When** the user completed the health assessment at the first time
**Then** the report in the admin dashboard is updated.
**When** the user completed the health assessment right after the first time.
**Then** The report in the admin dashboard is updated once again.
**And** the old report is replaced by the new one with new time.

Remarks: The duration between first assessment and second assessment is within five minutes.
Input: Sii Jia Wei
Device: Windows 10 Google Chrome Telegram Web

Tested by: Jia Wei

### 1.10.1.2 Scenario : Doctor remarks has been added

**Given** the user completed the health assessment twice in a day.
**When** the user completed the health assessment at the first time
**And** the doctor adds his remarks on
**When** the user updates his health assessment again
**Then** The doctor remarks still persists.

Remarks: Results within expectations

Tested by: Si Tian

# 1.11 Non-Reported Table

## 1.11.1 Feature : Update Non-Reported Table

### 1.11.1.1 Scenario : Update everyday at 12.00am

Given the user did a health assessment on the first day.
And the data has been recorded in Reports.
When the time has come to 12.00am the next day
Then the data should be moved to “Non-reported”
But the data was not moved to “Non-reported”
And the data is still at the “Reports”

Input: Sii Jia Wei
NRIC in admin dashboard: 970202020202
Date of first health assessment: 5 April 2020
Device: Windows 10 Google Chrome Telegram Web
Remarks: This is the second health assessment which was done on 5 April. It means that it has replaced the first health assessment done on the same day.

| Submitted at | NRIC/Passport | Patient name | Patient phone number | Calling status | Cough | Throat | Fever | Breathe | Chest | Blue | Drowsy | Has symptom | Patient's Remark | Docto |
|---|---|---|---|---|---|---|---|---|---|---|---|---|---|---|
| 05/04/2020, 02:09:07 TODAY | 930515065147 | CHEN XIANG | 60169363909 | No call yet | No cough | Start having sore throat today | No fever | No breathing difficulty | No chest pain | No blue face | No drowsy | ✓ | | |
| 05/04/2020, 01:17:28 TODAY | 12121212 | YOW | 60199904589 | Don't have to call | No cough | No sore throat | No fever | No breathing difficulty | No chest pain | No blue face | No drowsy | ✗ | | |
| 05/04/2020, 01:02:35 TODAY | 960101084321 | TEST_KHOOI | 60165366568 | Don't have to call | No cough | No sore throat | No fever | No breathing difficulty | No chest pain | No blue face | No drowsy | ✗ | | |
| 05/04/2020, 00:43:51 TODAY | 1442 | BRANDON | 60129118018 | No call yet | No changes | No sore throat | No fever | No breathing difficulty | Has chest pain | No blue face | Has drowsy | ✓ | | |
| 04/04/2020, 23:28:39 | 788788 | CHAN CHEE SENG | 60166213998 | No call yet | No changes | Getting better | No fever | No breathing difficulty | Has chest pain | No blue face | No drowsy | ✓ | 海底捞好吃吗？可是DrLiew只喜欢吃lok2..然后Dr Chiew要求每人需要有一公里那么长的距... | 能这[illegible] 看来[illegible] 装的。 底捞[illegible] hmmn |
| 04/04/2020, 22:35:01 | 1234556666 | CHIN HAI | 60179192669 | No call yet | No changes | No sore throat | No changes | No breathing difficulty | No chest pain | No blue face | No drowsy | ✓ | | |
| 04/04/2020, 20:24:46 | 970705565210 | TMY | 601135979877 | Don't have to call | No cough | No sore throat | No fever | No breathing difficulty | No chest pain | No blue face | No drowsy | ✗ | https://stackoverflow.com/questions/1592534/what-i... | |
| 04/04/2020, 18:53:26 | 999 | XIONG TEST REPORT | 60127153013 | Don't have to call | No changes | No changes | No changes | No breathing difficulty | No chest pain | No blue face | No drowsy | ✗ | | |
| 04/04/2020, 18:40:32 | 970202020202 | SII JIA WEI | 60138274493 | Don't have to call | No cough | No sore throat | No fever | No breathing difficulty | No chest pain | No blue face | No drowsy | ✗ | test \n \n \n test test | |
| 04/04/2020, 16:54:52 | 909090 | TRY | 60199904587 | No call yet | Start coughing today | No sore throat | No changes | No breathing difficulty | No chest pain | No blue face | No drowsy | ✓ | | |
| 04/04/2020, 16:22:14 | ASD | ^%$ | 60178547588 | Don't have to call | No changes | No sore throat | No fever | No breathing difficulty | No chest pain | No blue face | No drowsy | ✗ | | |
| 04/04/2020, 15:11:26 | 999999999999 | ISSAC99 | 60134844797 | No call yet | Start coughing today | No sore throat | Start having a fever today | No breathing difficulty | Has chest pain | No blue face | Has drowsy | ✓ | | |
| | | | | | | Start | - | | | | | | | |

| | | | | | | | | | | | | | |
|---|---|---|---|---|---|---|---|---|---|---|---|---|---|
| 04/04/2020, 18:40:32 | 970202020202 | SII JIA WEI | 60138274493 | Don't have to call | No cough | No sore throat | No fever | No breathing difficulty | No chest pain | No blue face | No drowsy | ✗ | test \n \n \n test test |

This image is taken from “Reports”. It is supposed to be moved to “Non-Reported”.

# 1.12 Patient Table

## 1.12.1 Feature : Only One Phone Number Allowed

### 1.12.1.1 Scenario : Create a patient with existing phone number

**Given** there is a patient created with a phone number.
**When** another patient is created with the same phone number but different name, status and preferred language.
**Then** a toast message appears "Phone number already exists. Please use another phone number."

Remarks:
1. Bug tested.

Case Input 1:
1. NRIC: 965233652
2. Name: TT
3. Phone number: 0138274493
4. Status: Asymptomatic
5. Preferred language: English

Case Input 2:
1. NRIC: 85236241TY
2. Name: TT90
3. 0138274493
4. Symptomatic
5. Malay

Tested by JiaWei

## 1.12.2 Feature : Preferred Language

### 1.12.2.1 Scenario : Admin changes preferred language after the patient has finished a health assessment

**Given** the patient's default language is English
**And** the patient has finished a health assessment
**When** the admin changes the language to Mandarin
**And** the patient starts to do health assessment
**Then** the health assessment is in Mandarin
**When** the admin changes the language to Malay
**Then** the health assessment is in Malay

Remark:
1. Result expected

Tested by jiawei

# 1.12.3 Feature : Patient status sorting

## 1.12.3.1 Scenario : Admin sorts the status of patient

**Given** the admin wants to sort the status of patient to see those who has quit or passed away

**When** the admin clicks on the “Status” of the patient

**And** now those who have passed away are at the end of the table

**And** the admin clicks “Next” to the end

**Then** the “passed away” cases should be bonded together

**But there is one “Recovered” in between them**

**When** the admin clicks again on the “Status” of the patient

**And** now those who have passed away are at the beginning of the table

**Then** the “passed away” cases should be grouped together

**But there is one “Recovered” case in between them**

Remarks: Only “Passed away” or “Quit” has such an issue.

Reference:

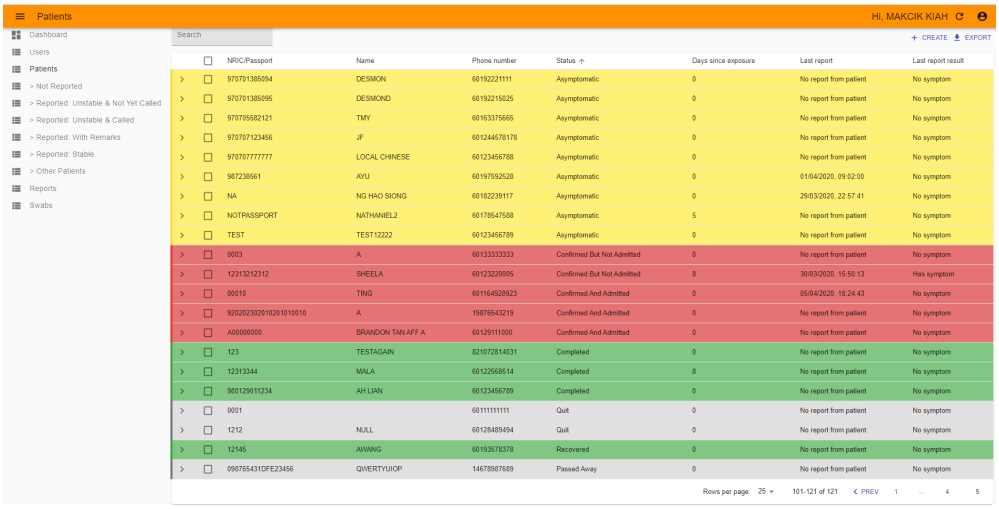


| NRIC/Passport | Name | Phone number | Status ↑ | Days since exposure | Last report | Last report result |
|---|---|---|---|---|---|---|
| 970701385094 | DESMON | 60192221111 | Asymptomatic | 0 | No report from patient | No symptom |
| 970701385095 | DESMOND | 60192215025 | Asymptomatic | 0 | No report from patient | No symptom |
| 970705582121 | TMY | 60163375665 | Asymptomatic | 0 | No report from patient | No symptom |
| 970707123456 | JF | 601244578178 | Asymptomatic | 0 | No report from patient | No symptom |
| 970707777777 | LOCAL CHINESE | 60123456788 | Asymptomatic | 0 | No report from patient | No symptom |
| 987238561 | AYU | 60197592528 | Asymptomatic | 0 | 01/04/2020, 09:02:00 | No symptom |
| NA | NG HAO SIONG | 60182239117 | Asymptomatic | 0 | 29/03/2020, 22:57:41 | No symptom |
| NOTPASSPORT | NATHANIEL2 | 60178547588 | Asymptomatic | 5 | No report from patient | No symptom |
| TEST | TEST12222 | 60123456789 | Asymptomatic | 0 | No report from patient | No symptom |
| 0003 | A | 60133333333 | Confirmed But Not Admitted | 0 | No report from patient | No symptom |
| 12313212312 | SHEELA | 60123220005 | Confirmed But Not Admitted | 8 | 30/03/2020, 15:50:13 | Has symptom |
| 00010 | TING | 601164928923 | Confirmed And Admitted | 0 | 05/04/2020, 18:24:43 | No symptom |
| 92020230201020101010010 | A | 19876543219 | Confirmed And Admitted | 0 | No report from patient | No symptom |
| A00000000 | BRANDON TAN AFF A | 60129111000 | Confirmed And Admitted | 0 | No report from patient | No symptom |
| 123 | TESTAGAIN | 821072814031 | Completed | 0 | No report from patient | No symptom |
| 12313344 | MALA | 60122568514 | Completed | 8 | No report from patient | No symptom |
| 980129011234 | AH LIAN | 60123456789 | Completed | 0 | No report from patient | No symptom |
| 0001 | | 60111111111 | Quit | 0 | No report from patient | No symptom |
| 1212 | NULL | 60128489494 | Quit | 0 | No report from patient | No symptom |
| 12145 | AWANG | 60193578378 | Recovered | 0 | No report from patient | No symptom |
| 098765431DFE23456 | QWERTYUIOP | 14678987689 | Passed Away | 0 | No report from patient | No symptom |

Rows per page: 25 101-121 of 121 PREV 1 ... 4 5

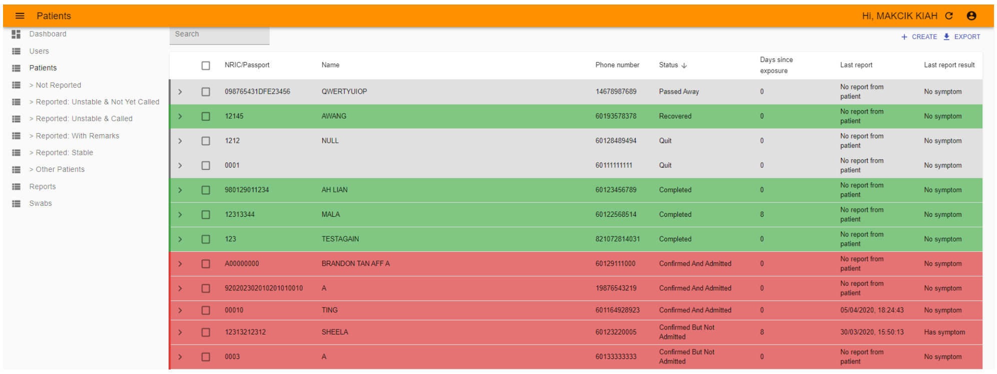


| NRIC/Passport | Name | Phone number | Status ↓ | Days since exposure | Last report | Last report result |
|---|---|---|---|---|---|---|
| 098765431DFE23456 | QWERTYUIOP | 14678987689 | Passed Away | 0 | No report from patient | No symptom |
| 12145 | AWANG | 60193578378 | Recovered | 0 | No report from patient | No symptom |
| 1212 | NULL | 60128489494 | Quit | 0 | No report from patient | No symptom |
| 0001 | | 60111111111 | Quit | 0 | No report from patient | No symptom |
| 980129011234 | AH LIAN | 60123456789 | Completed | 0 | No report from patient | No symptom |
| 12313344 | MALA | 60122568514 | Completed | 8 | No report from patient | No symptom |
| 123 | TESTAGAIN | 821072814031 | Completed | 0 | No report from patient | No symptom |
| A00000000 | BRANDON TAN AFF A | 60129111000 | Confirmed And Admitted | 0 | No report from patient | No symptom |
| 92020230201020101010010 | A | 19876543219 | Confirmed And Admitted | 0 | No report from patient | No symptom |
| 00010 | TING | 601164928923 | Confirmed And Admitted | 0 | 05/04/2020, 18:24:43 | No symptom |
| 12313212312 | SHEELA | 60123220005 | Confirmed But Not Admitted | 8 | 30/03/2020, 15:50:13 | Has symptom |
| 0003 | A | 60133333333 | Confirmed But Not Admitted | 0 | No report from patient | No symptom |

### 1.12.4 Feature : Create Patient with different preferred language

#### 1.12.4.1 Scenario : Create Patient with different preferred language

Given an admin is creating a patient
When the admin chooses English for preferred language
And the admin clicks on “Save” button
Then the patient is created on table with correct preferred language
When the admin chooses Chinese for preferred language
And the admin clicks on “Save” button
Then the patient is created on table with correct preferred language
When the admin chooses Malay for preferred language
And the admin clicks on “Save” button
Then the patient is created on table with correct preferred language

Remark: Result Expected
Tested by JiaWei

## 1.12.3 Feature : Adding new patient

### 1.12.3.1 Scenario : Insert patient name with various 'special' Unicode characters

**Given** a new patient record is created with name
**When** <input> is inserted in name field
**Then** save button is clicked

Test Case:
*Ignore ‘<’ and ‘>’

| Case | Unicode/Type of characters | <input> | Valid/Invalid | Remarks |
|---|---|---|---|---|
| 1 | U+0020 Space | < > | Invalid | |
| 2 | U+00A0 No-Break Space | < > | Invalid | `sout('\u00a0');` |
| 3 | U+2000 En Quad | <  > | Invalid | |
| 4 | U+2001 Em Quad | <   > | Invalid | |
| 5 | U+2002 En Space | <  > | Invalid | |
| 6 | U+2003 Em Space | <   > | Invalid | |
| 7 | U+2004 Three-Per-Em Space | < > | Invalid | |
| 8 | U+2005 Four-Per-Em Space | < > | Invalid | |
| 9 | U+2006 Six-Per-Em Space | < > | Invalid | |
| 10 | U+2007 Figure Space | <  > | Invalid | |
| 11 | U+2008 Punctuation Space | < > | Invalid | |
| 12 | U+2009 Thin Space | < > | Invalid | |
| 13 | U+200A Hair Space | <> | Invalid | |

| | | | | |
|---|---|---|---|---|
| 14 | U+200B Zero Width Space | <> | Invalid | |
| 15 | U+200C Zero Width Non-Joiner | <> | Invalid | |
| 16 | U+200D Zero Width Joiner | <> | Invalid | |
| 17 | U+200E Left-To-Right Mark | <> | Invalid | |
| 18 | U+200F Right-To-Left Mark | <> | Invalid | |
| 19 | U+2028 Line Separator | <[SEP]> | Invalid | `sout('\u2028');` |
| 20 | U+202A Left-To-Right Embedding | <> | Invalid | |
| 21 | U+202B Right-To-Left Embedding | <> | Invalid | |
| 22 | U+202C Pop Directional Formatting | <> | Invalid | |
| 23 | U+202D Left-To-Right Override | <> | Invalid | |
| 24 | U+202E Right-To-Left Override | << | Invalid | |
| 25 | U+202F Narrow No-Break Space | < > | Invalid | |
| 26 | U+205F Medium Mathematical Space | < > | Invalid | `sout('\u205f');` |
| 27 | U+2063 Invisible Separator | <[,]> | Invalid | `sout('\u2063');` |
| 28 | U+206A Inhibit Symmetric Swapping | <> | Invalid | |
| 29 | U+206B Activate Symmetric Swapping | <> | Invalid | |
| 30 | U+206C Inhibit Arabic Form Shaping | <> | Invalid | |

| | | | | |
|---|---|---|---|---|
| 31 | U+206D Activate Arabic Form Shaping | <> | Invalid | |
| 32 | U+206E National Digit Shapes | <> | Invalid | |
| 33 | U+206F Nominal Digit Shapes | <> | Invalid | |
| 34 | U+2800 Braille Pattern Blank | < > | Invalid | ('\u2800'); |
| 35 | U+3000 Ideographic Space | <　> | Invalid | |
| 36 | U+FEFF Zero Width No-Break Space | <> | Invalid | ('\ufeff'); |
| 37 | U+FF10 Full Width Digit Zero | ０ ０ ０ ０ ０ ０ ０ ０ | Valid | ('\uff10'); |
| 38 | Chinese | 病人 | Valid | |
| 39 | Zalgo Text | ABC | Invalid | |
| 40 | Zalgo Text (Less Character) | ABC | Valid | Ignored for now, the damage dealt by less character is less |
| 41 | Superscript | PATIENT | Valid | |
| 42 | Subscript | patient | Valid | |

Remarks:
*(Display nothing after save)*

| | | NRIC/Passport ↑ | Name | Phone number | Status | Days |
|---|---|---|---|---|---|---|
| › | ☐ | 0001 | | 60111111111 | Not Applicable | 0 |

How to produce Unicode in Win10

1. Press Start button
2. Search “Character Map” and hit enter
3. Find the symbol you want
4. Click “Select”
5. Click “Copy”

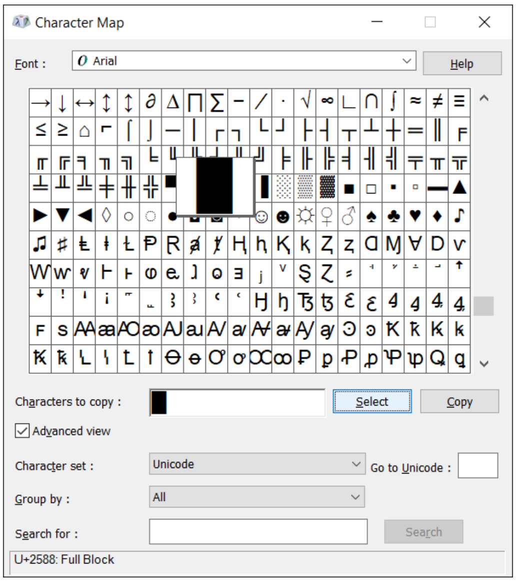


Zalgo Text: https://lingojam.com/GlitchTextGenerator
Superscript & subscript: https://lingojam.com/TinyTextGenerator

Tested by TING WEI JING
Update 7/4/2020 6:34pm

## 1.12.3 Feature : Bot verdict, dashboard symptoms and days since fever from Asymptomatic Patient

### 1.12.3.1 Scenario : Asymptomatic patient takes health assessment with only one “Yes”

Given the patient is set as asymptomatic at the beginning
When the patient takes health assessment
Then the patient should get correct bot verdict
And the dashboard should record correct symptoms
And the days since first fever should be correct

Test Cases:

1. All No

| | |
|---|---|
| Cough | No |
| Sore Throat | No |
| Fever | No |
| Difficulty in breathing | No |
| Chest pain | No |
| Face/ Lips Turning blue | No |
| Drowsy | No |
| Other symptoms? | No |
| **Date: 11/4/20 \| Date of first fever: 9/4/20 \| Initial Days Since First Fever: 3** | |
| Bot Verdict | Stay home |
| Dashboard symptoms | Matched |
| Days since first fever | 3 |
| **Date: 11/4/20 \| Date of first fever: 10/4/20 \| Initial Days Since First Fever: 2** | |
| Bot Verdict | Stay home |
| Dashboard symptoms | Matched |
| Days since first fever | 2 |
| **Date: 11/4/20 \| Date of first fever: - \| Initial Days Since First Fever: 0** | |
| Bot Verdict | Stay home |
| Dashboard symptoms | Matched |
| Days since first fever | 0 |

2. All “Yes”

| | | | | | | | | |
|---|---|---|---|---|---|---|---|---|
| Cough | Yes | | | | | | | |
| Sore Throat | | Yes | | | | | | |
| Fever | | | Yes | | | | | |
| Difficulty in Breathing | | | | Yes | | | | |
| Chest Pain | | | | | Yes | | | |
| Face/Lips turning blue | | | | | | Yes | | |
| Drowsy | | | | | | | Yes | |
| Other symptoms? | | | | | | | | Yes |
| **Date: 11/4/20 \| Date of first fever: 9/4/20 \| Initial Days Since First Fever: 3** | | | | | | | | |
| Bot Verdict | Cond chg – call ummc | Cond chg – call ummc | Cond chg – call ummc | Cond chg – call ummc | Cond chg – call ummc | Cond chg – call ummc | Cond chg – call ummc | Stay home |
| Dashboard symptoms | Matched hasSym | Matched hasSym | Matched hasSym | Matched hasSym | Matched hasSym | Matched hasSym | Matched hasSym | Matched noSym |
| Days since first fever | 3 | 3 | 3 | 3 | 3 | 3 | 3 | 3 |
| **Date: 11/4/20 \| Date of first fever: 10/4/20 \| Initial Days Since First Fever: 2** | | | | | | | | |
| Bot Verdict | Cond chg – call ummc | Cond chg – call ummc | Cond chg – call ummc | Cond chg – call ummc | Cond chg – call ummc | Cond chg – call ummc | Cond chg – call ummc | Stay home |
| Dashboard symptoms | Matched hasSym | Matched hasSym | Matched hasSym | Matched hasSym | Matched hasSym | Matched hasSym | Matched hasSym | Matched noSym |
| Days since first fever | 2 | 2 | 2 | 2 | 2 | 2 | 2 | 2 |
| **Date: 11/4/20 \| Date of first fever: - \| Initial Days Since First Fever: 0** | | | | | | | | |
| Bot Verdict | Cond chg – call ummc | Cond chg – call ummc | Cond chg – call ummc | Cond chg – call ummc | Cond chg – call ummc | Cond chg – call ummc | Cond chg – call ummc | Stay home |
| Dashboard symptoms | Matched hasSym | Matched hasSym | Matched hasSym | Matched hasSym | Matched hasSym | Matched hasSym | Matched hasSym | Matched noSym |
| Days since first fever | 0 | 0 | 1 | 0 | 0 | 0 | 0 | 0 |

Tested by jiawei

### 1.12.3.1 Scenario : Asymptomatic patient takes health assessment with two “Yes”

Given the patient is set as asymptomatic at the beginning
When the patient takes health assessment
Then the patient should get correct bot verdict
And the dashboard should record correct symptoms
And the days since first fever should be correct

Test Cases:

1. Cough

| | | | | | | | |
|---|---|---|---|---|---|---|---|
| Cough | Yes | Yes | Yes | Yes | Yes | Yes | Yes |
| Sore Throat | Yes | | | | | | |
| Fever | | Yes | | | | | |
| Difficulty in Breathing | | | Yes | | | | |
| Chest Pain | | | | Yes | | | |
| Face/Lips turning blue | | | | | Yes | | |
| Drowsy | | | | | | Yes | |
| Other symptoms? | | | | | | | Yes |
| **Date: 11/4/20 \| Date of first fever: 9/4/20 \| Initial Days Since First Fever: 3** | | | | | | | |
| Bot Verdict | Cond chg – call ummc | Cond chg – call ummc | Cond chg – call ummc | Cond chg – call ummc | Cond chg – call ummc | Cond chg – call ummc | Cond chg – call ummc |
| Dashboard symptoms | Matched hasSym | Matched hasSym | Matched hasSym | Matched hasSym | Matched hasSym | Matched hasSym | Matched hasSym |
| Days since first fever | 3 | 3 | 3 | 3 | 3 | 3 | 3 |
| **Date: 11/4/20 \| Date of first fever: 10/4/20 \| Initial Days Since First Fever: 2** | | | | | | | |
| Bot Verdict | Cond chg – call ummc | Cond chg – call ummc | Cond chg – call ummc | Cond chg – call ummc | Cond chg – call ummc | Cond chg – call ummc | Cond chg – call ummc |
| Dashboard symptoms | Matched hasSym | Matched hasSym | Matched hasSym | Matched hasSym | Matched hasSym | Matched hasSym | Matched hasSym |
| Days since first fever | 2 | 2 | 2 | 2 | 2 | 2 | 2 |
| **Date: 11/4/20 \| Date of first fever: - \| Initial Days Since First Fever: 0** | | | | | | | |
| Bot Verdict | Cond chg – call ummc | Cond chg – call ummc | Cond chg – call ummc | Cond chg – call ummc | Cond chg – call ummc | Cond chg – call ummc | Cond chg – call ummc |
| Dashboard symptoms | Matched hasSym | Matched hasSym | Matched hasSym | Matched hasSym | Matched hasSym | Matched hasSym | Matched hasSym |
| Days since first fever | 0 | 1 | 0 | 0 | 0 | 0 | 0 |

2. Sore throat

| | | | | | | | |
|---|---|---|---|---|---|---|---|
| Cough | Yes | | | | | | |
| Sore Throat | Yes | Yes | Yes | Yes | Yes | Yes | Yes |
| Fever | | Yes | | | | | |
| Difficulty in Breathing | | | Yes | | | | |
| Chest Pain | | | | Yes | | | |
| Face/Lips turning blue | | | | | Yes | | |
| Drowsy | | | | | | Yes | |
| Other symptoms? | | | | | | | Yes |
| **Date: 11/4/20 \| Date of first fever: 9/4/20 \| Initial Days Since First Fever: 3** | | | | | | | |
| Bot Verdict | Cond chg – call ummc | Cond chg – call ummc | Cond chg – call ummc | Cond chg – call ummc | Cond chg – call ummc | Cond chg – call ummc | Cond chg – call ummc |
| Dashboard symptoms | Matched hasSym | Matched hasSym | Matched hasSym | Matched hasSym | Matched hasSym | Matched hasSym | Matched hasSym |
| Days since first fever | 3 | 3 | 3 | 3 | 3 | 3 | 3 |
| **Date: 11/4/20 \| Date of first fever: 10/4/20 \| Initial Days Since First Fever: 2** | | | | | | | |
| Bot Verdict | Cond chg – call ummc | Cond chg – call ummc | Cond chg – call ummc | Cond chg – call ummc | Cond chg – call ummc | Cond chg – call ummc | Cond chg – call ummc |
| Dashboard symptoms | Matched hasSym | Matched hasSym | Matched hasSym | Matched hasSym | Matched hasSym | Matched hasSym | Matched hasSym |
| Days since first fever | 2 | 2 | 2 | 2 | 2 | 2 | 2 |
| **Date: 11/4/20 \| Date of first fever: - \| Initial Days Since First Fever: 0** | | | | | | | |
| Bot Verdict | Cond chg – call ummc | Cond chg – call ummc | Cond chg – call ummc | Cond chg – call ummc | Cond chg – call ummc | Cond chg – call ummc | Cond chg – call ummc |
| Dashboard symptoms | Matched hasSym | Matched hasSym | Matched hasSym | Matched hasSym | Matched hasSym | Matched hasSym | Matched hasSym |
| Days since first fever | 0 | 1 | 0 | 0 | 0 | 0 | 0 |

3. Fever

| | | | | | | | |
|---|---|---|---|---|---|---|---|
| Cough | Yes | | | | | | |
| Sore Throat | | Yes | | | | | |
| Fever | Yes | Yes | Yes | Yes | Yes | Yes | Yes |
| Difficulty in Breathing | | | Yes | | | | |
| Chest Pain | | | | Yes | | | |
| Face/Lips turning blue | | | | | Yes | | |
| Drowsy | | | | | | Yes | |
| Other symptoms? | | | | | | | Yes |
| **Date: 11/4/20 \| Date of first fever: 9/4/20 \| Initial Days Since First Fever: 3** | | | | | | | |
| Bot Verdict | Cond chg – call ummc | Cond chg – call ummc | Cond chg – call ummc | Cond chg – call ummc | Cond chg – call ummc | Cond chg – call ummc | Cond chg – call ummc |
| Dashboard symptoms | Matched hasSym | Matched hasSym | Matched hasSym | Matched hasSym | Matched hasSym | Matched hasSym | Matched hasSym |
| Days since first fever | 3 | 3 | 3 | 3 | 3 | 3 | 3 |
| **Date: 11/4/20 \| Date of first fever: 10/4/20 \| Initial Days Since First Fever: 2** | | | | | | | |
| Bot Verdict | Cond chg – call ummc | Cond chg – call ummc | Cond chg – call ummc | Cond chg – call ummc | Cond chg – call ummc | Cond chg – call ummc | Cond chg – call ummc |
| Dashboard symptoms | Matched hasSym | Matched hasSym | Matched hasSym | Matched hasSym | Matched hasSym | Matched hasSym | Matched hasSym |
| Days since first fever | 2 | 2 | 2 | 2 | 2 | 2 | 2 |
| **Date: 11/4/20 \| Date of first fever: - \| Initial Days Since First Fever: 0** | | | | | | | |
| Bot Verdict | Cond chg – call ummc | Cond chg – call ummc | Cond chg – call ummc | Cond chg – call ummc | Cond chg – call ummc | Cond chg – call ummc | Cond chg – call ummc |
| Dashboard symptoms | Matched hasSym | Matched hasSym | Matched hasSym | Matched hasSym | Matched hasSym | Matched hasSym | Matched hasSym |
| Days since first fever | 1 | 1 | 1 | 1 | 1 | 1 | 1 |

4. Difficulty in breathing

| | | | | | | | |
|---|---|---|---|---|---|---|---|
| Cough | Yes | | | | | | |
| Sore Throat | | Yes | | | | | |
| Fever | | | Yes | | | | |
| Difficulty in Breathing | Yes | Yes | Yes | Yes | Yes | Yes | Yes |
| Chest Pain | | | | Yes | | | |
| Face/Lips turning blue | | | | | Yes | | |
| Drowsy | | | | | | Yes | |
| Other symptoms? | | | | | | | Yes |
| **Date: 11/4/20 \| Date of first fever: 9/4/20 \| Initial Days Since First Fever: 3** | | | | | | | |
| Bot Verdict | Cond chg – call ummc | Cond chg – call ummc | Cond chg – call ummc | Cond chg – call ummc | Cond chg – call ummc | Cond chg – call ummc | Cond chg – call ummc |
| Dashboard symptoms | Matched hasSym | Matched hasSym | Matched hasSym | Matched hasSym | Matched hasSym | Matched hasSym | Matched hasSym |
| Days since first fever | 3 | 3 | 3 | 3 | 3 | 3 | 3 |
| **Date: 11/4/20 \| Date of first fever: 10/4/20 \| Initial Days Since First Fever: 2** | | | | | | | |
| Bot Verdict | Cond chg – call ummc | Cond chg – call ummc | Cond chg – call ummc | Cond chg – call ummc | Cond chg – call ummc | Cond chg – call ummc | Cond chg – call ummc |
| Dashboard symptoms | Matched hasSym | Matched hasSym | Matched hasSym | Matched hasSym | Matched hasSym | Matched hasSym | Matched hasSym |
| Days since first fever | 2 | 2 | 2 | 2 | 2 | 2 | 2 |
| **Date: 11/4/20 \| Date of first fever: - \| Initial Days Since First Fever: 0** | | | | | | | |
| Bot Verdict | Cond chg – call ummc | Cond chg – call ummc | Cond chg – call ummc | Cond chg – call ummc | Cond chg – call ummc | Cond chg – call ummc | Cond chg – call ummc |
| Dashboard symptoms | Matched hasSym | Matched hasSym | Matched hasSym | Matched hasSym | Matched hasSym | Matched hasSym | Matched hasSym |
| Days since first fever | 0 | 0 | 1 | 0 | 0 | 0 | 0 |

5. Chest Pain

| | | | | | | | |
|---|---|---|---|---|---|---|---|
| Cough | Yes | | | | | | |
| Sore Throat | | Yes | | | | | |
| Fever | | | Yes | | | | |
| Difficulty in Breathing | | | | Yes | | | |
| Chest Pain | Yes | Yes | Yes | Yes | Yes | Yes | Yes |
| Face/Lips turning blue | | | | | Yes | | |
| Drowsy | | | | | | Yes | |
| Other symptoms? | | | | | | | Yes |
| **Date: 11/4/20 \| Date of first fever: 9/4/20 \| Initial Days Since First Fever: 3** | | | | | | | |
| Bot Verdict | Cond chg – call ummc | Cond chg – call ummc | Cond chg – call ummc | Cond chg – call ummc | Cond chg – call ummc | Cond chg – call ummc | Cond chg – call ummc |
| Dashboard symptoms | Matched hasSym | Matched hasSym | Matched hasSym | Matched hasSym | Matched hasSym | Matched hasSym | Matched hasSym |
| Days since first fever | 3 | 3 | 3 | 3 | 3 | 3 | 3 |
| **Date: 11/4/20 \| Date of first fever: 10/4/20 \| Initial Days Since First Fever: 2** | | | | | | | |
| Bot Verdict | Cond chg – call ummc | Cond chg – call ummc | Cond chg – call ummc | Cond chg – call ummc | Cond chg – call ummc | Cond chg – call ummc | Cond chg – call ummc |
| Dashboard symptoms | Matched hasSym | Matched hasSym | Matched hasSym | Matched hasSym | Matched hasSym | Matched hasSym | Matched hasSym |
| Days since first fever | 2 | 2 | 2 | 2 | 2 | 2 | 2 |
| **Date: 11/4/20 \| Date of first fever: - \| Initial Days Since First Fever: 0** | | | | | | | |
| Bot Verdict | Cond chg – call ummc | Cond chg – call ummc | Cond chg – call ummc | Cond chg – call ummc | Cond chg – call ummc | Cond chg – call ummc | Cond chg – call ummc |
| Dashboard symptoms | Matched hasSym | Matched hasSym | Matched hasSym | Matched hasSym | Matched hasSym | Matched hasSym | Matched hasSym |
| Days since first fever | 0 | 0 | 1 | 0 | 0 | 0 | 0 |

6. Face/Lips Turning Blue

| | | | | | | | |
|---|---|---|---|---|---|---|---|
| Cough | Yes | | | | | | |
| Sore Throat | | Yes | | | | | |
| Fever | | | Yes | | | | |
| Difficulty in Breathing | | | | Yes | | | |
| Chest Pain | | | | | Yes | | |
| Face/Lips turning blue | Yes | Yes | Yes | Yes | Yes | Yes | Yes |
| Drowsy | | | | | | Yes | |
| Other symptoms? | | | | | | | Yes |
| **Date: 11/4/20 \| Date of first fever: 9/4/20 \| Initial Days Since First Fever: 3** | | | | | | | |
| Bot Verdict | Cond chg – call ummc | Cond chg – call ummc | Cond chg – call ummc | Cond chg – call ummc | Cond chg – call ummc | Cond chg – call ummc | Cond chg – call ummc |
| Dashboard symptoms | Matched hasSym | Matched hasSym | Matched hasSym | Matched hasSym | Matched hasSym | Matched hasSym | Matched hasSym |
| Days since first fever | 3 | 3 | 3 | 3 | 3 | 3 | 3 |
| **Date: 11/4/20 \| Date of first fever: 10/4/20 \| Initial Days Since First Fever: 2** | | | | | | | |
| Bot Verdict | Cond chg – call ummc | Cond chg – call ummc | Cond chg – call ummc | Cond chg – call ummc | Cond chg – call ummc | Cond chg – call ummc | Cond chg – call ummc |
| Dashboard symptoms | Matched hasSym | Matched hasSym | Matched hasSym | Matched hasSym | Matched hasSym | Matched hasSym | Matched hasSym |
| Days since first fever | 2 | 2 | 2 | 2 | 2 | 2 | 2 |
| **Date: 11/4/20 \| Date of first fever: - \| Initial Days Since First Fever: 0** | | | | | | | |
| Bot Verdict | Cond chg – call ummc | Cond chg – call ummc | Cond chg – call ummc | Cond chg – call ummc | Cond chg – call ummc | Cond chg – call ummc | Cond chg – call ummc |
| Dashboard symptoms | Matched hasSym | Matched hasSym | Matched hasSym | Matched hasSym | Matched hasSym | Matched hasSym | Matched hasSym |
| Days since first fever | 0 | 0 | 1 | 0 | 0 | 0 | 0 |

7. Drowsy

| | | | | | | | |
|---|---|---|---|---|---|---|---|
| Cough | Yes | | | | | | |
| Sore Throat | | Yes | | | | | |
| Fever | | | Yes | | | | |
| Difficulty in Breathing | | | | Yes | | | |
| Chest Pain | | | | | Yes | | |
| Face/Lips turning blue | | | | | | Yes | |
| Drowsy | Yes | Yes | Yes | Yes | Yes | Yes | Yes |
| Other symptoms? | | | | | | | Yes |
| **Date: 11/4/20 \| Date of first fever: 9/4/20 \| Initial Days Since First Fever: 3** | | | | | | | |
| Bot Verdict | Cond chg – call ummc | Cond chg – call ummc | Cond chg – call ummc | Cond chg – call ummc | Cond chg – call ummc | Cond chg – call ummc | Cond chg – call ummc |
| Dashboard symptoms | Matched hasSym | Matched hasSym | Matched hasSym | Matched hasSym | Matched hasSym | Matched hasSym | Matched hasSym |
| Days since first fever | 3 | 3 | 3 | 3 | 3 | 3 | 3 |
| **Date: 11/4/20 \| Date of first fever: 10/4/20 \| Initial Days Since First Fever: 2** | | | | | | | |
| Bot Verdict | Cond chg – call ummc | Cond chg – call ummc | Cond chg – call ummc | Cond chg – call ummc | Cond chg – call ummc | Cond chg – call ummc | Cond chg – call ummc |
| Dashboard symptoms | Matched hasSym | Matched hasSym | Matched hasSym | Matched hasSym | Matched hasSym | Matched hasSym | Matched hasSym |
| Days since first fever | 2 | 2 | 2 | 2 | 2 | 2 | 2 |
| **Date: 11/4/20 \| Date of first fever: - \| Initial Days Since First Fever: 0** | | | | | | | |
| Bot Verdict | Cond chg – call ummc | Cond chg – call ummc | Cond chg – call ummc | Cond chg – call ummc | Cond chg – call ummc | Cond chg – call ummc | Cond chg – call ummc |
| Dashboard symptoms | Matched hasSym | Matched hasSym | Matched hasSym | Matched hasSym | Matched hasSym | Matched hasSym | Matched hasSym |
| Days since first fever | 0 | 0 | 1 | 0 | 0 | 0 | 0 |

Tested by jiawei

# 1.13 Dashboard Line Graph

## 1.13.1 Feature : Details

### 1.13.1.1 Scenario : Line graph title

Given a line graph consists of numerical data
And user wants to check for the date
And the date comes out in form of XX/XX/XX to XX/XX/XX

**Remarks: Results within expectations**
Tested by: Si Tian

# 1.14 Fever Cont Days

## 1.14.1 Feature : Days with fever

### 1.14.1.1 Scenario : First day of getting fever

Given there were no first date of fever recorded
And this is the first day of getting fever
When the user completes the health assessment
Then the bot should answer “You are stable. Stay at home…”
And the days since first fever should be “1”
But the bot answers “Your status has changed…”
And the days since the first fever is 0.

Remark:

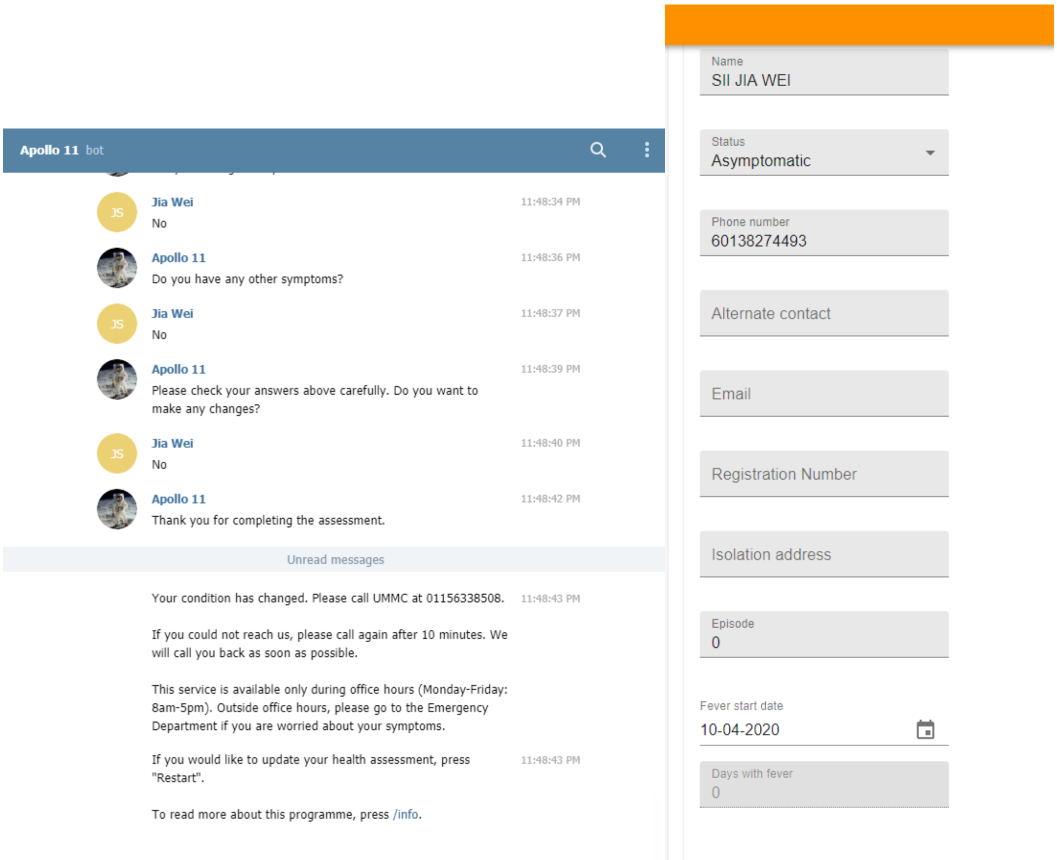


Tested by: Jia Wei.

# 2.0 Telegram Bot

## 2.1 Health Assessment

### 2.1.1 Feature : Taking health assessment

#### 2.1.1.1 Scenario : User uses two test bots

**Given** user has linked his phone number to UMMC database
**And** user creates a group chat named "Group" in Telegram with UMMC Test bot
**And** user himself in this group chat (2 members only)
**When** user selects UMMC Test bot to chat
**Then** user presses "Start" button
**When** UMMC Test bot asks "*Do you have any cough?*"
**And** "Yes" and "No" buttons appear
**Then** user presses back button to return to Telegram main interface
**Then** user selects "Group" to enter the group chat which created early
**Then** user types "/help"
**And** send message
**When** user receives "*... To start a new health assessment, press "Start"*" in group chat
**Then** user presses "Start"
**And** user presses back button to return to Telegram main interface
**Then** user selects UMMC Test bot to chat again
**Then** user presses "No"
**But** UMMC Test bot replies "*I don't understand what you have responded.*"
**Then** user presses "Yes"
**But** UMMC Test bot replies "*An error has occurred. Please try again. To read more about this programme, press /info.*"
**Then** user presses "/info"
**Then** user presses "/help"
**End**

**Remark 1:**

Unexpected result

Tested using Huawei Y9 2019 Android 8.1.0
Telegram Version 5.15.0

Tested using Win 10
Telegram Desktop Version 1.9.14
Name: TING
Phone Number: 01164928923

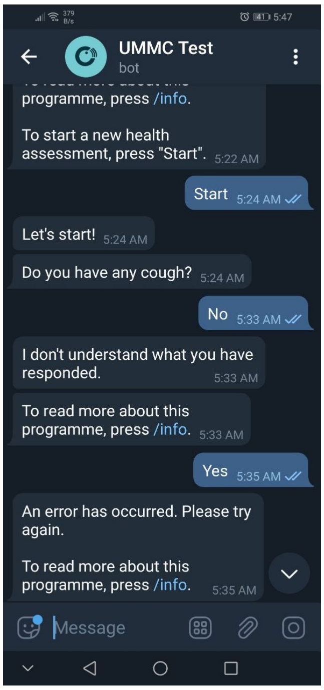


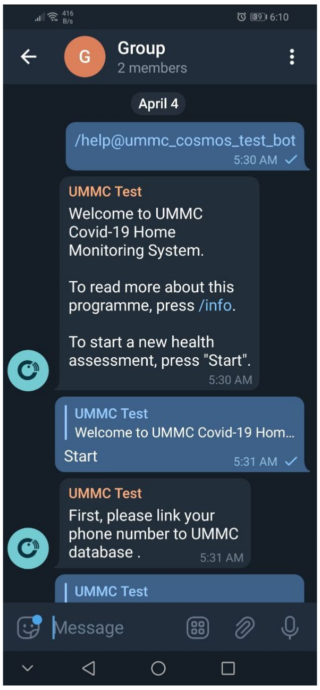


**Remark 2 (not based on scenario 2.1.1.1) :**

Unexpected result

Elaboration of actions to achieve the output:

1. Goes to patient dashboard to delete existing patient account
2. Then test if the bot can still allow assessment or not
3. Good expected output as bot does not allow assessment with message “Tool is for UMMC patients…”
4. Leaves phone for few hours and not replying bot
5. Returns to dashboard and create a new patient to test again.
6. Returns back to telegram to chat with bot and start the conversation with “Start”
7. Bot responds with “I don’t understand what you have responded “ even though the expected output from “Start” should direct to “link phone number”.

Phone : iPhone Xr
IOS version : 13.3.1
Telegram version : 6.0.1
Name : Lee Dywei
Contact Number : 0182824297

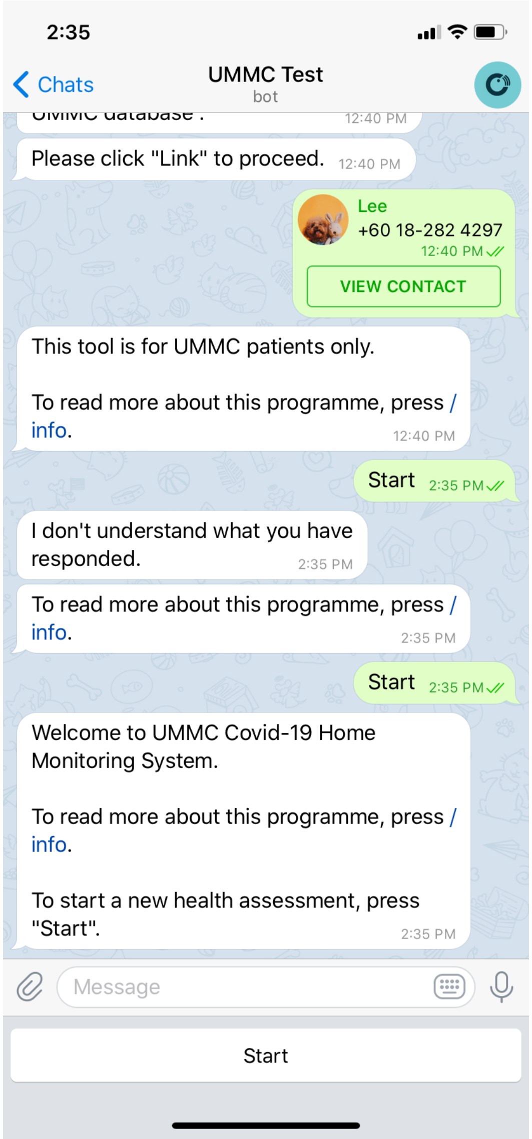


### 2.1.1.3 Scenario : Patient responds with a sticker/GIF (Bug has been fixed)

**Given** patient has linked his phone number to the db
**When** the bot asks about the health conditions
**And** patient replies with a sticker / GIF
**But bot gives no response**

Remarks: Expected results: Bot replies “I don’t understand what you have responded.”
Name: Yow Si Tian
PhoneNumber: 0199904589
Tested using Redmi 3S AndroPERFORMANCE ISSUE: REWRITE THE WHOLE BOT, DUMP TELEGRAM BOT APIid 6.0.1

### 2.1.1.4 Scenario : Accessing bot using unregistered number

**Given** user has not linked his/her phone number to UMMC database
**And** user initiates Telegram Desktop testbot and gets told “This tool is for UMMC patients only…” after 1 standard flow
**Then** user continues with /info
**Then** Link instead of /help -> Gets an “I don’t understand…” message
**Then** /help
**When** “To read more about this programme, press /info.” message comes out.
**Then** Start
**When** shown the message “Welcome...press “Start””
**But** the same message “Welcome...press “Start”.” shows up again
**Then** Start
**Then** press Link
**Then** gets restricted as number is not registered

Remarks:
Expected: No duplicate message pop up according to flow

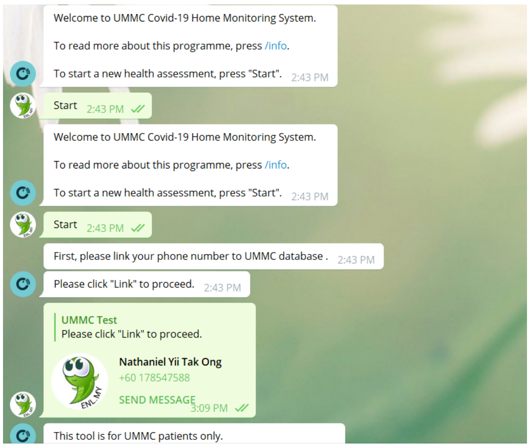


Tested using Windows 10 Home
Telegram Desktop Version 2.0.1
Name: Nat
Phone Number: -

### 2.1.1.5 Scenario : Accessing additional information for guidance during Q&A session

**Given** user has linked his/her phone number to UMMC database
**And** Start
**Then** user switched to keyboard, ignoring the options to be chosen
**And** user tried to obtain available guidance by typing the /info or /help options
**Then** the corresponding messages of /info or /help popped up
**Then** user looked for the options panel to continue the answering session
**But** the option panel now only shows “Start”
**Then** Start
**Then** user has to answer everything from the beginning

Remarks:
Expected: User can continue answering after clearing his/her mind by reading the assisting documents.

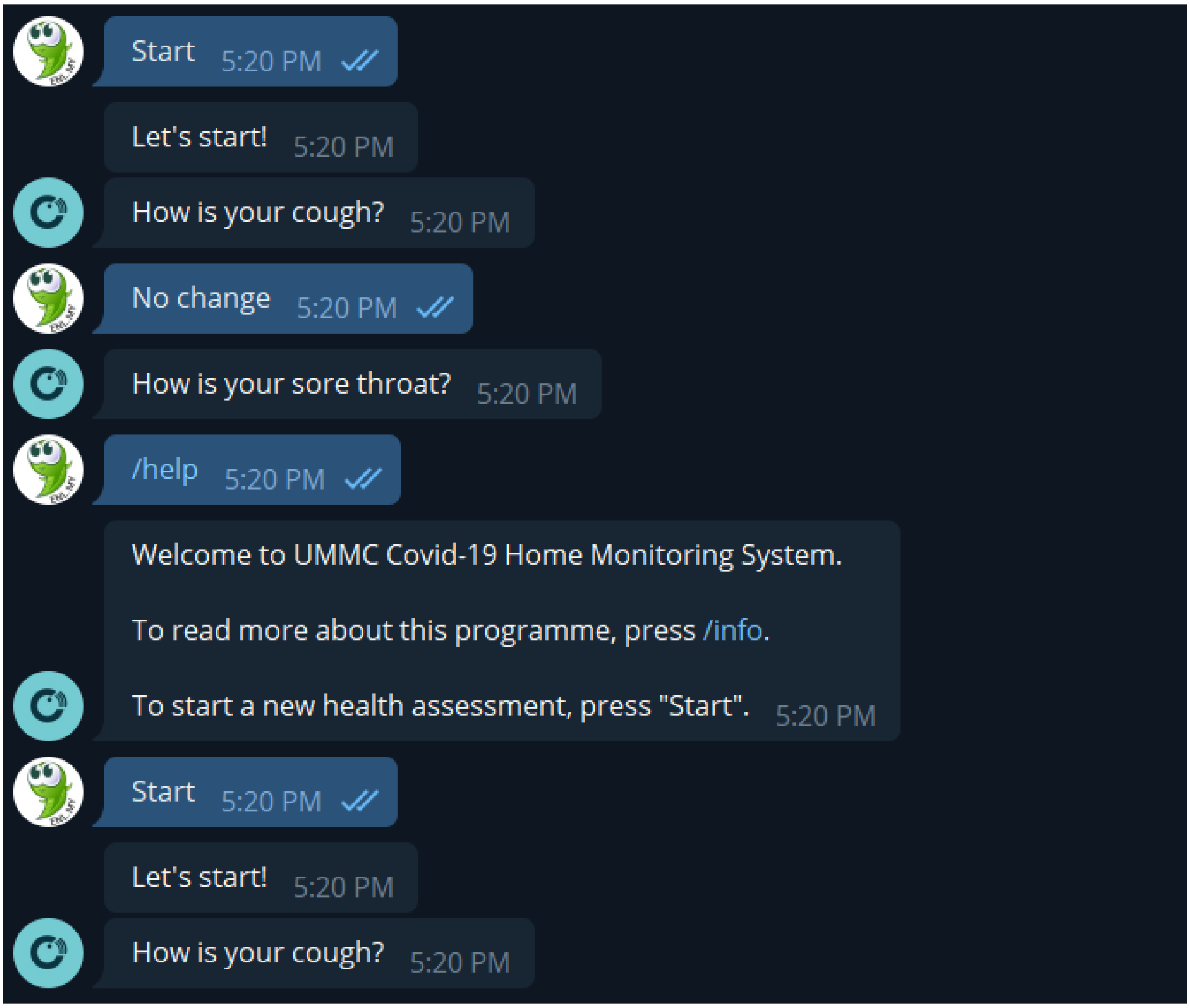


Tested using Windows 10 Home
Telegram Desktop Version 2.0.1
Name: Nat
Phone Number: 60178547588

### 2.1.1.6 **Scenario** : Bot in a group

**Given** user added the bot into a group deliberately
**And** type /start deliberately
**When** user click/type the "start button" as usual
**Then** the bot answered "First,please link your phone number to…."
**And** did not show the "share your contact number notification"

Remarks: When the user replied to the bot message, the bot answered "I don't understand..." as usual

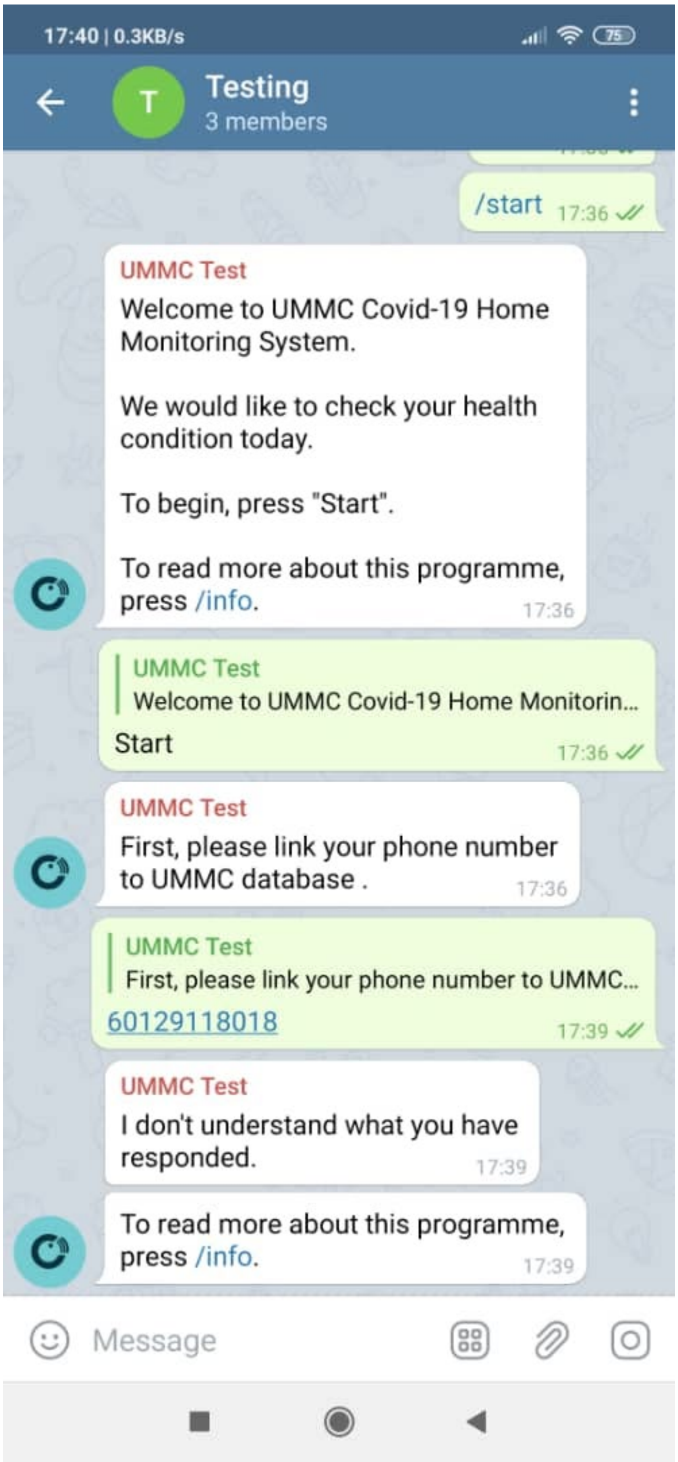


Tested using Redmi Note 7
Name: Brandon
Phone Number: 60129118018

### 2.1.1.7 Scenario : Accessing personal information

**Given** user has linked his/her phone number to UMMC database
**And** tries to look for commands to obtain personal details like name
**But** user fails to find any

Remarks:
Expected: User can access and check his/her details through a certain command that is discoverable to the user.

Tested using Windows 10 Home
Telegram Desktop Version 2.0.1
Name: Nat
Phone Number: 60178547588

#### **2.1.1.8 Scenario** : User cleared/deleted his history chat

**Given** user has linked his phone number to the db
**When** the user cleared/deleted the history chat
**Then** bot bypassed the contact number and give health assessment as usual

Tested using Redmi Note 7
Name: Brandon
PhoneNumber: 0129118018

#### 2.1.1.9 Scenario: Spamming on the bot with "No"

**Given** the user is given access to health assessment
**When** the user starts the assessment
**And** the user spams on a question with several "No"
**Then** the bot should reply "Please press once only" first, only continue with the following questions.
**And** the bot should have ignored all the repeating "No".
**And** the bot should have only come out with one question "Do you have a sore throat?" at a time.
**But** the bot replies "Please press once only" after questions are coming out.
**And** the bot comes out with two questions at the same time
**And** the bot only receives one "No" to two questions, then proceeds to the next question.

Remarks: All the issues happen simultaneously when people spams on it.
Input: Only "No"
Device: Telegram Web, Google Chrome
Tested by: Jia Wei
Reference:

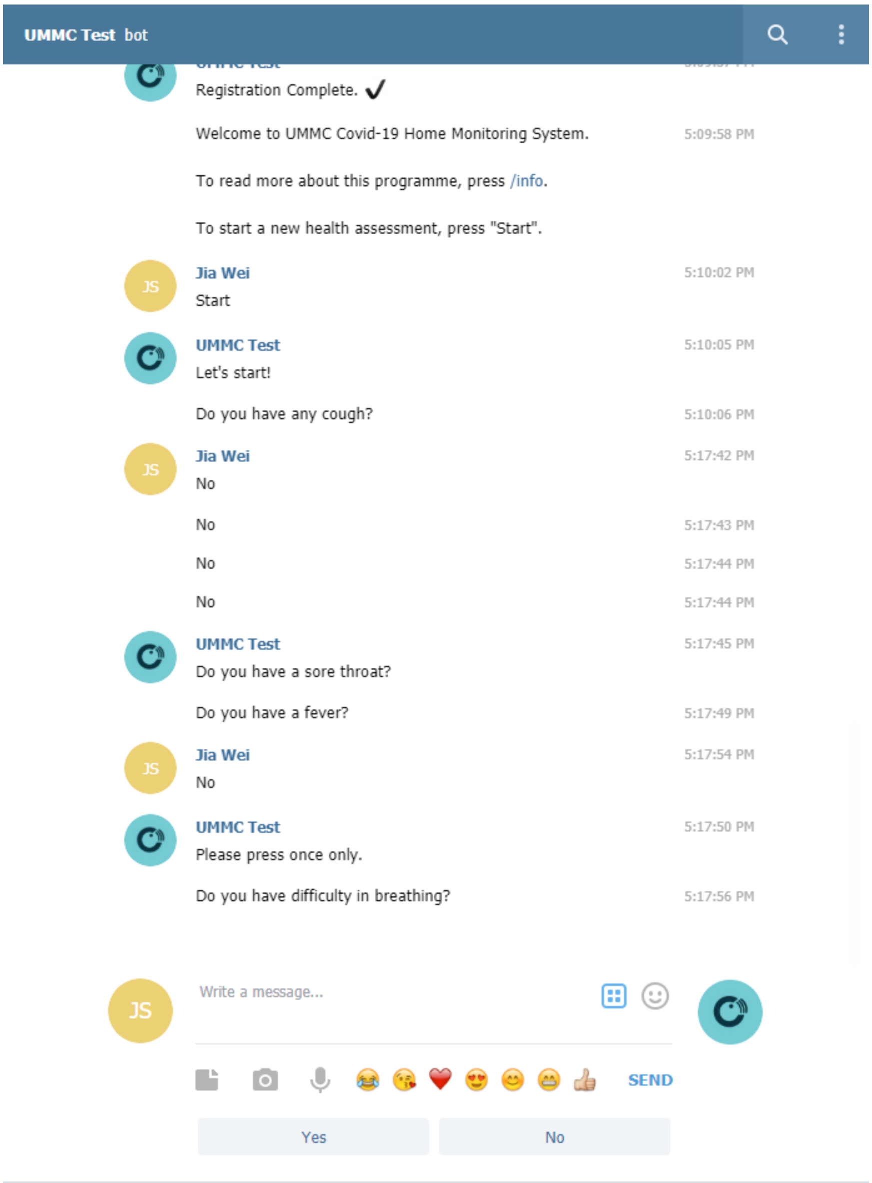


### 2.1.1.10 Scenario : User accidentally choose the wrong answer on one question

**Given** user has linked his/her phone number to UMMC database
**And** answer a question wrongly
**But** the user has to answer the rest of the question until the "Please check your answers..." question pops up.

Remarks:
Expected: A command/ message/ inline keyboard to allow the user to answer the same question again before continue to answer the rest of the question

Tested using Redmi Note 7
Name: Brandon
Phone Number: 60129118018

**2.1.1.11 Scenario** :Other symptoms data collection

**Given** user click yes for “Do you have other symptoms?”
**And** trying to key in the other symptoms
**When** user key in the symptoms all in one message
**And** send it
**And** want to add on certain other symptoms he/she miss out to mentioned in the previous message,no add on can be done.
**Then** the message on’ Please check your answers above carefully.Do you want to make any changes ‘is replied .

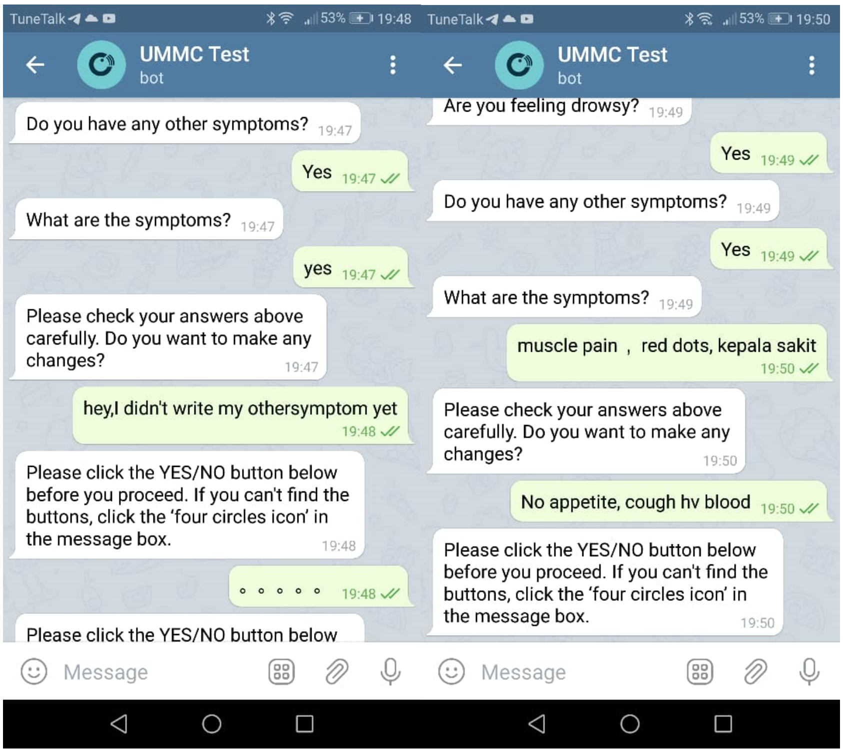


Remarks:Key in on other symptoms must be done in one message.User cannot separately replied in more than one message regarding the other symptoms .To make the add on of other symptoms he/she miss out,user to restart the assessment again.
Expected: A command/ message/ inline keyboard to allow the user to answer the same question again or to confirm they already complete filling in the other symptoms before continue to the next question.

Tested using Huaweip9
Phone Number: 601135970877

Tested by: Mei Yih

### 2.1.1.12 Scenario : User responds by forwarding messages

**Given** user has linked his phone number to UMMC database
**And** user sends “Yes”, “No”, “Yes”, “No”, “Msg 1”, “Msg 2”, “Msg 3”, “Yes”, “No” and “Msg 4” in Saved Messages (see Remarks for details)
**When** user starts health assessment with UMMC test bot
**Then** bot asks “*Do you have any cough?*”
**Then** user forwards “Yes” message from Saved Messages to bot
**Then** bot asks “*Do you have a sore throat?*”
**Then** user forwards “Yes” and “No” messages at the same time to bot
**Then** bot replies “*Please press once only.*”
**And** asks “*Do you have a fever?*”
**Then** user forwards “Yes”, “No” and “Yes” messages to bot
**Then** bot replies “*Please press once only.*”
**And** asks “*Do you have difficulty in breathing?*”
**Then** user forwards “Yes”, “No”, “Yes”, “No”, “Msg 1”, “Msg 2”and “Msg 3” to bot
**Then** bot replies “*Please press once only.*”
**And** asks “*Do you have chest pain?*”
**Then** user forwards “Msg 1”, “Msg 2”, “Msg 3”, “Yes” and “No” to bot
**Then** bot replies “*Please click the YES/NO button below...*”
**And** “*Please press once only.*”
**Then** user forwards “Msg 3”, “Yes”, “No” and “Msg 4” to bot
**Then** bot replies “*Please click the YES/NO button below...*”
**And** “*Please press once only.*”
**Then** user forwards “No” message to bot
**Until** bot asks “*Do you have any other symptoms?*”
**Then** user answers “Yes”
**Then** bot asks “*What are the symptoms?*”
**Then** user forwards “Msg 1”, “Msg 2” and “Msg 3” to bot
**But** bot replies “*Please press once only.*”
**Then** bot replies “*Please check your answers above carefully. Do you want to make any changes?*”
**Then** user answer “No”
End

**Remark:**
**Checked collected data, UMMC test bot only accepts first forwarded message.**

| Cough | Throat | Fever | Breathe | Chest | Blue | Drowsy | Has symptom | Remarks |
|---|---|---|---|---|---|---|---|---|
| Start coughing today | Start having sore throat today | Start having a fever today | Has breathing difficulty | No chest pain | No blue face | No drowsy | ✓ | Msg 1 |

Tested using Huawei Y9 2019 Android 8.1.0
Telegram Version 5.15.0

Tested using Win 10
Telegram Desktop Version 1.9.14
Name: TING
Phone Number: 01164928923
Tested by: TING WEI JING

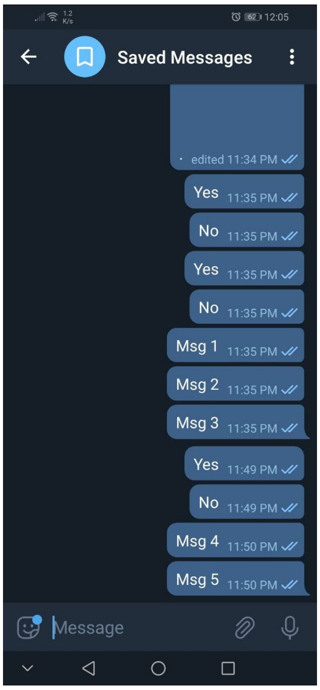


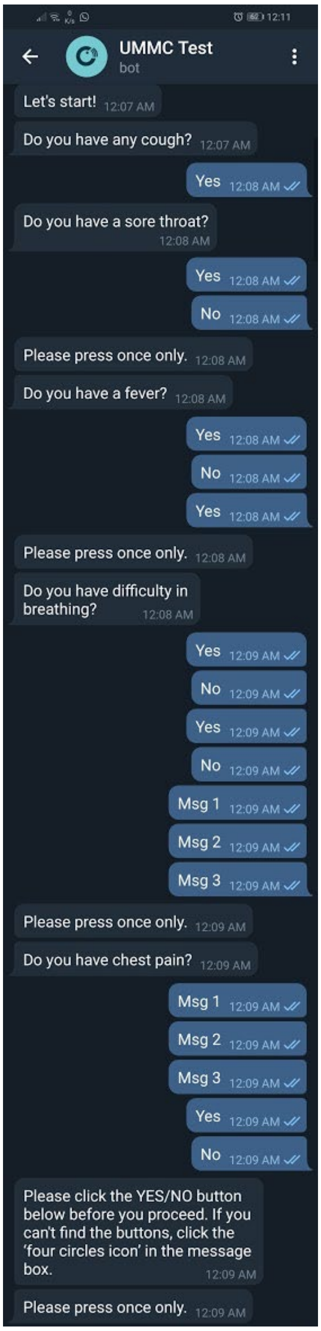


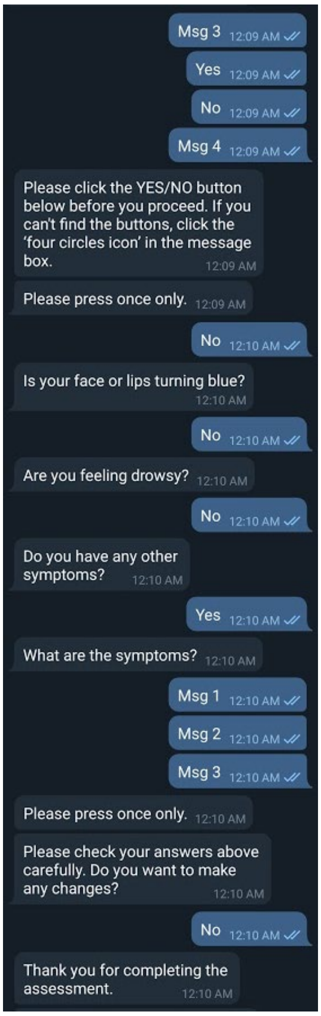


**2.1.1.13 Scenario** : User answered a Mandarin question using English(Yes/No)

**Given** user has linked his phone number to the db

**When** the user answered a Mandarin question using “Yes”
**But** bot did not reply anything

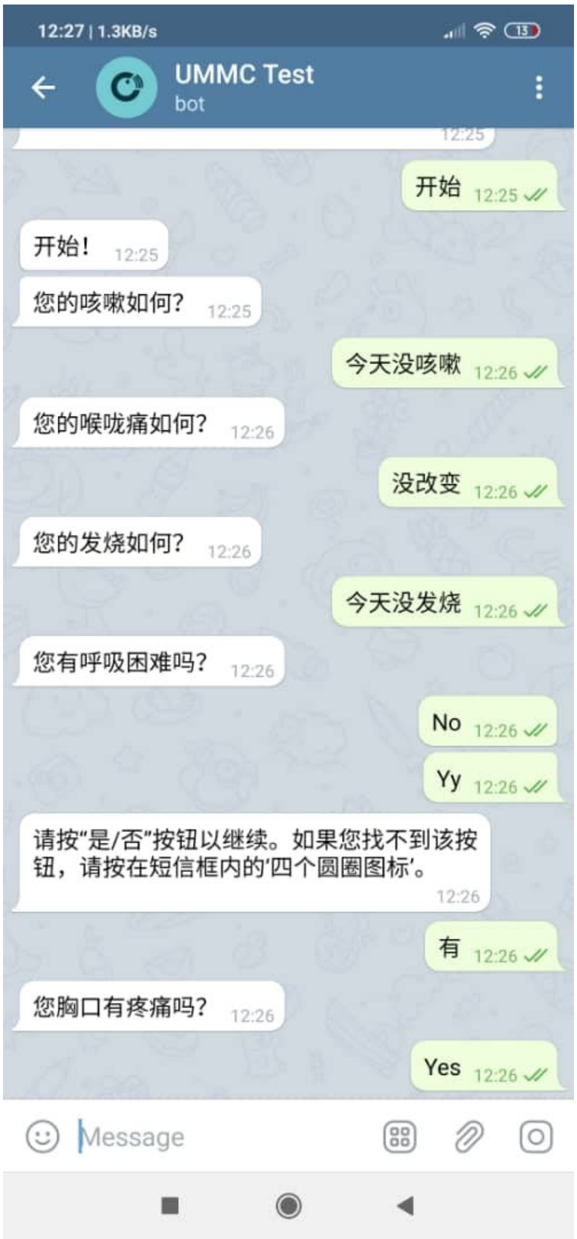


Tested using Redmi Note 7
Name: Brandon
PhoneNumber: 0129118018
**Refer to Scenario 2.1.1.17, by Jason and Issac**

Remark 2: Bug solved and retested by Jia Wei

### 2.1.1.14 **Scenario** : User maliciously tries to link other phone numbers

**Given** user A and B has registered their phone number in DB
**And** malicious user has their phone number but does not know if they are a patient or not
**When** bot ask to link your phone number to db
**And** malicious user forwards other people's number instead
**Then** bot will reply "An error has occurred, Please try again"

**Remark:**
Working as expected. The malicious user will have no way to find out if user A and B are patients or not.
This ONLY applies if user A and B has submitted a report at least once.

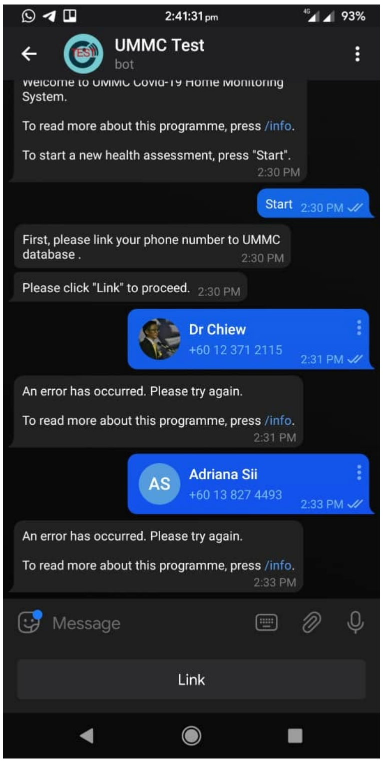


| | NRIC/Passport | Name | Phone number | Status | Days since exposure | Last report ↓ |
|---|---|---|---|---|---|---|
| > | 012345678901 | CHIEW THIAM KIAN | 60123712115 | Symptomatic | 0 | 05/04/2020, 12:24:35 TODAY |

| | NRIC/Passport | Name | Phone number | Status | Days since exposure | Last report ↓ |
|---|---|---|---|---|---|---|
| > | 970202020202 | SII JIA WEI | 60138274493 | Asymptomatic | 2 | 04/04/2020, 18:40:32 |

Tested using Xiaomi Mi A2, Android 10 (March 2020 Security Patch)
Telegram version 6.0.1
Name: Jason

### **2.1.1.15 Scenario** : Patients deleted Telegram chats.

**Given** the patient has completed health assessment using Telegram Web
**And** the patient did not exit the Telegram web
**And** after that the patient deleted the Telegram chats using phone
**And** the patient did not search back the bot using phone
**And** the patient is not deleted from the admin dashboard
**Then** the content of the Telegram Web is cleared.
**And** only left a “Start” button
**When** the patient came back to Telegram Web that he/she did not exit in the beginning
**And** the patient clicked on the “Start” button
**Then** it should let the patient to start the health assessment
**But** Error occurred. “One of the params is missing or invalid.”

Remark:

1. It can be solved by searching back the bot from the phone and starting the bot using the phone, then when coming back to Telegram Web, everything will go back to normal.

Input Device: Windows 10 Google Chrome, Android 8 Huawei nova 2

Reference

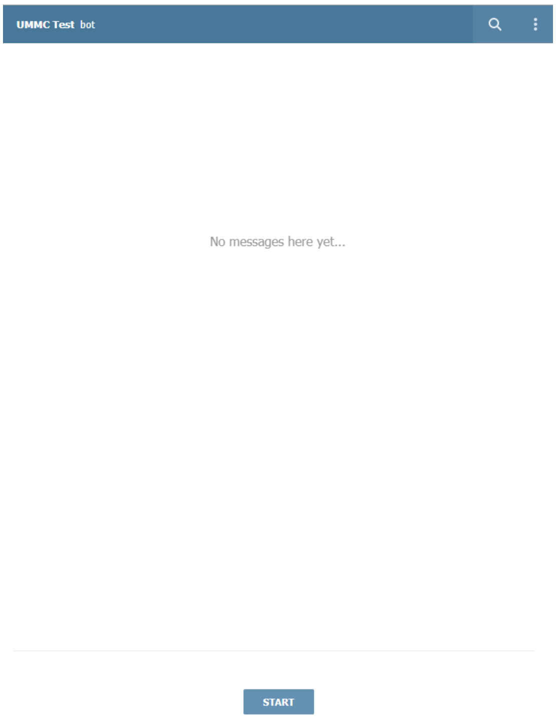

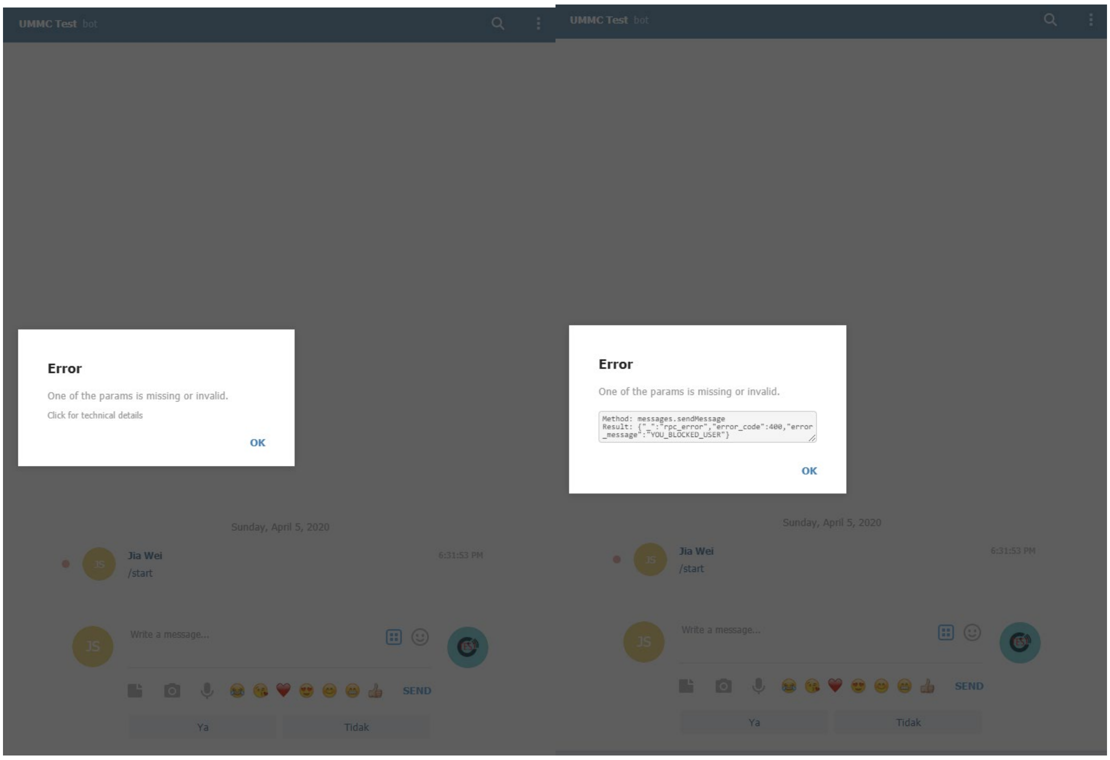


Tested by Jiawei.

### **2.1.1.16 Scenario** : Patient clicks on “Yes” and “No” button at the same time

Given the patient is doing health assessment using Telegram Web and Telegram App at the same time

When the patient presses “Yes” using Telegram App

And the patient clicks on “No” using Telegram Web

Then the bot answers “Mari kita ulang semula penilaian di atas.”

Remark: Testing result expected.
Tested by Jia Wei.

### **2.1.1.17 Scenario** : User answers the questions in a different language

**Given** user’s language preference is Malay
**When** bot asks a question regarding the symptom
**And** user replies in Chinese instead
**Then** bot will reply “Sila tekan butang...” error message as expected.
**But bot proceed to next question without letting the user to reanswer.**
**When** user submit the report to the bot
**But any skipped questions remains empty inside the report**

**Remark:**
Upon further testing it applies to all other languages too. Any words that exist in the choices (both asymptomatic and symptomatic) in other languages will cause the bot to reply a error message, then skip to the next question, but does not put anything into the report.

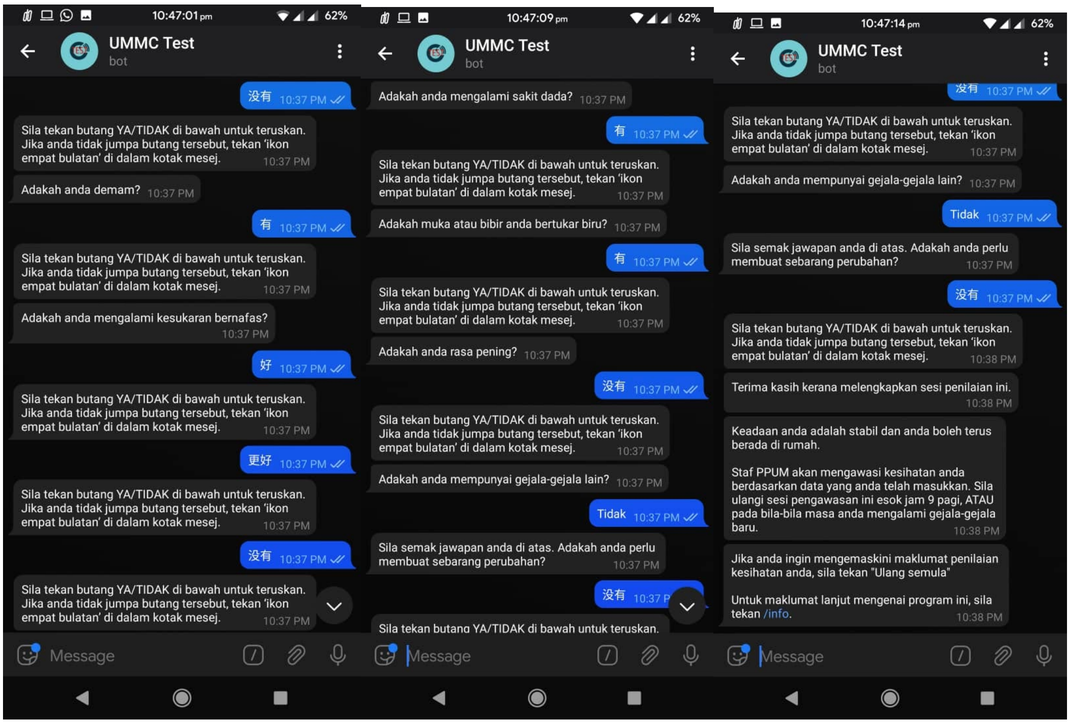


DETAILS | REPORTS | SWABS

| Submitted at ↓ | Calling status | Cough | Throat | Fever | Breathe | Chest | Blue | Drowsy |
|---|---|---|---|---|---|---|---|---|
| 05/04/2020, 22:38:01 TODAY | Don't have to call | | | | | | | |

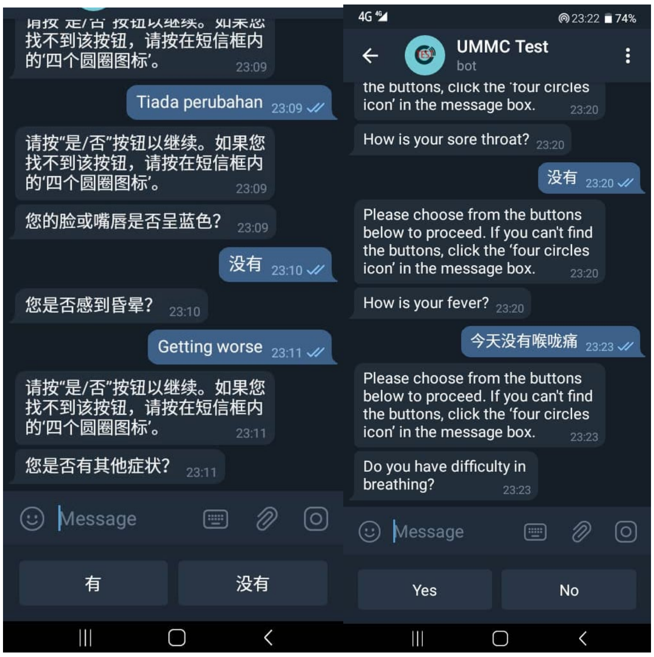


| Submitted at ↓ | Calling status | Cough | Throat | Fever | Breathe | Chest | Blue | Drowsy | Has symptom |
|---|---|---|---|---|---|---|---|---|---|
| 05/04/2020, 23:13:43 TODAY | Don't have to call | | | | | | No blue face | | ✕ |
| 04/04/2020, 15:11:26 | No call yet | Start coughing today | No sore throat | Start having a fever today | No breathing difficulty | Has chest pain | No blue face | Has drowsy | ✓ |
| 03/04/2020, 19:20:07 | UMMC called patient | Start coughing today | No sore throat | Start having a fever today | Has breathing difficulty | Has chest pain | Has blue face | No drowsy | ✓ |

Tested using:

Xiaomi Mi A2, Android 10 (March 2020 Security Patch)
Telegram version 6.0.1

Samsung A70, Android 8.1
Telegram version 6.0.1
Name: Jason and Issac

## 2.2 Notifications of Health Assessment

### 2.2.1 Feature : Receive Notification Every Morning

#### 2.2.1.1 Scenario : Taking Health Assessment Everyday

**Given** the user has done the health assessment
**When** the user is supposed to receive notification the next day
**But** no notification is received

Remark: It might be related to the scenario in 1.10.
Input name: Sii Jia Wei
Input NRIC: 970202020202
Input date and time: 04.04.2020 6.40pm
Device: Huawei nova 2i, Android 8.0

### 2.2.1.2 Scenario : Late Notification For Taking Health Assessment

**Given** the user has done the health assessment
**When** the user is supposed to receive notification the next day
**Then** the notification is received
**But** the notification is received late and multiple notifications are received within one hour

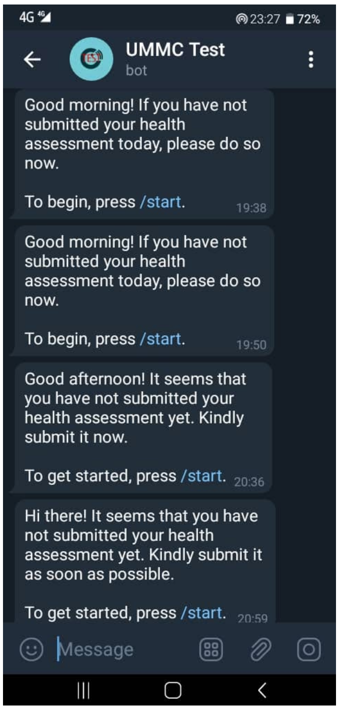


Remark:
Input name: Issac99
Input NRIC: 999999999999
Date: 5 Apr 2020 from 19:38 to 21:08
Device: Samsung A70, Android 8.1
Telegram Android 6.0.1

## 2.2.2 Feature : Receive Notification Of Prefered Language to Conduct Test

### 2.2.1.1 Scenario : Getting notification of preferred language when assessment for the day isn't done

**Given** the user has changed preferred language from Bahasa Malaysia to Chinese
**Then** the user receives nudging notifications in Chinese to prompt user to get assessment done.
**But** the button still shows instruction Bahasa Malaysia
**And** the instructions shown is “Restart” in English

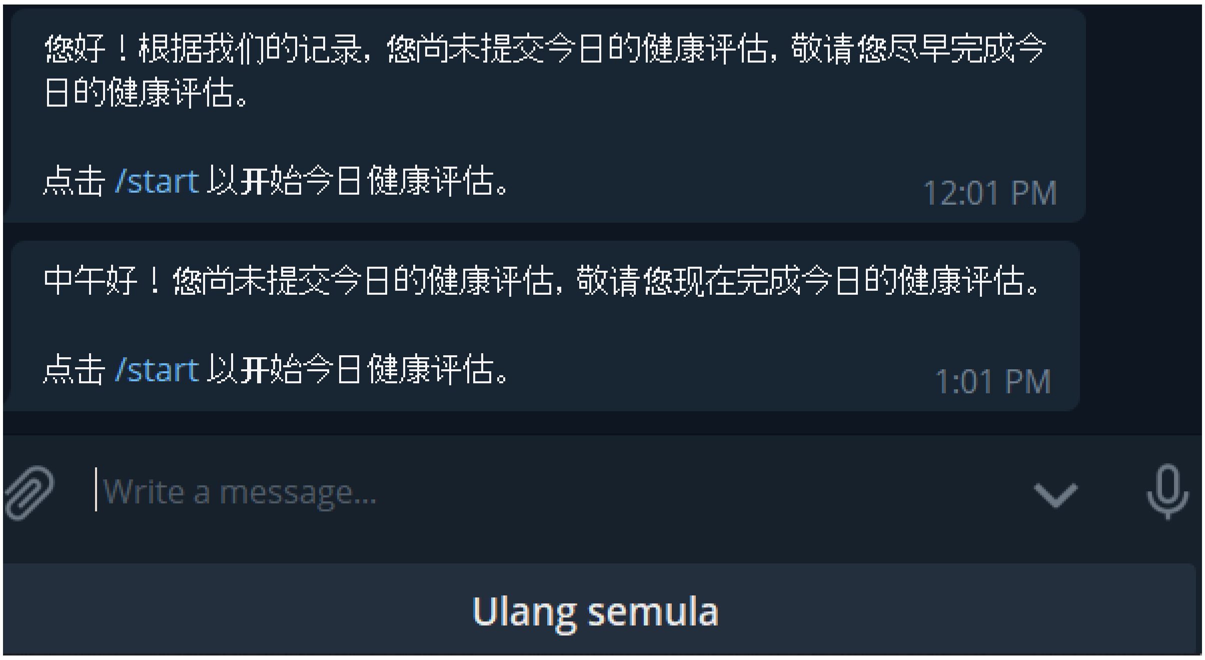


Remark: Language of button doesn't seem to synchronize with the prompting message notifications
Tested using Windows 10 Home
Telegram Desktop Version 2.0.1
Name: Nat
Phone Number: 60178547588
